# Cardiovascular–Kidney–Metabolic Syndrome and Health Risk Behaviors in U.S. Adults

**DOI:** 10.1016/j.focus.2026.100529

**Published:** 2026-07-15

**Authors:** Mehek Sharma, Darshan Chudasama, Biplab K. Datta, Steven S. Coughlin, Marlo M. Vernon, Jennifer C. Sullivan

**Affiliations:** 1 Medical College of Georgia, Augusta University, Augusta, Georgia; 2 Institute of Public and Preventive Health, School of Public Health, Augusta University, Augusta, Georgia; 3 Department of Health Management, Economics and Policy, School of Public Health, Augusta University, Augusta, Georgia; 4 Department of Biostatistics, Data Science and Epidemiology, School of Public Health, Augusta University, Augusta, Georgia; 5 Department of Physiology, Medical College of Georgia, Augusta University, Augusta, Georgia

**Keywords:** Alcohol consumption, cigarette smoking, vaping, cardiometabolic conditions, kidney disease

## Abstract

**Introduction:**

Cigarette smoking, vaping, and excessive alcohol consumption are established risk factors for many diseases, but few studies have examined these health-risk behaviors in relation to cardiovascular–kidney–metabolic syndrome. This study aims to assess the odds of current smoking, vaping, and heavy drinking by cardiovascular–kidney–metabolic syndrome stage and whether the odds vary by sex.

**Methods:**

This cross-sectional study used pooled data on 538,123 adults from the 2021 and 2023 waves of the Behavioral Risk Factor Surveillance System survey. Multivariable binomial logistic models stratified by sex and age group were estimated to assess the predicted probabilities of respective health risk behaviors for cardiovascular–kidney–metabolic syndrome stages, determined by self-reported health conditions. In addition, the interaction between sex and cardiovascular–kidney–metabolic syndrome stage was assessed to determine whether there were differences between males and females at different cardiovascular–kidney–metabolic syndrome stages.

**Results:**

The likelihood of current smoking and vaping was higher among those with an advanced cardiovascular–kidney–metabolic syndrome stage (i.e., 4) than among adults with lower cardiovascular–kidney–metabolic syndrome stages (i.e., 0, 1). Although males had a higher likelihood of smoking and vaping at cardiovascular–kidney–metabolic syndrome stages 0 and 1, no differences between males and females were found at cardiovascular–kidney–metabolic syndrome Stage 4. In contrast, the likelihood of heavy drinking was lower among females at cardiovascular–kidney–metabolic syndrome Stage 4 and higher among males at cardiovascular–kidney–metabolic syndrome Stages 2 and 3 than among those at lower cardiovascular–kidney–metabolic syndrome stages.

**Conclusions:**

Findings of this observational study suggest a higher likelihood of smoking and vaping among adults with advanced cardiovascular–kidney–metabolic syndrome stages, highlighting the scope for improving awareness about health-risk behaviors among patients to help them better manage their cardiovascular–kidney–metabolic syndrome conditions.

## HIGHLIGHTS

• Evidence on health-risk behaviors by cluster of conditions denoting cardiovascular–kidney–metabolic syndrome (CKMS) is scant.
• Current smoking and vaping are higher among those with advanced CKMS stages.
• Heavy drinking is lower in women but not in men at advanced CKMS stages.
• Smoking, vaping, and heavy drinking vary by CKMS stage across age groups and sexes.

## INTRODUCTION

Cardiovascular–kidney–metabolic syndrome (CKMS) is a recently defined health condition that acknowledges the interrelatedness of cardiovascular disease (CVD), kidney disease, and metabolic risk factors, all of which frequently co-occur and exacerbate one another, leading to multiorgan dysfunction and high rates of morbidity and mortality.^1^ The clinical consequences of CKMS are varied and complex. Individuals with CKMS exhibit an increased risk of CVD (e.g., myocardial injury, heart failure, or stroke), chronic kidney disease (CKD) progression, impaired glucose tolerance, and Type 2 diabetes.^1^ These intertwined disease pathways are mediated by shared mechanisms, including systemic inflammation, oxidative stress, neurohormonal activation, and endothelial dysfunction, which together drive multiorgan deterioration.^2^

Because these interrelated conditions often emerge and progress in the context of poor lifestyle habits, modifiable behavioral risk factors represent critical targets for prevention and management within the CKMS framework. Among these, cigarette smoking is a well-established contributor to a broad array of chronic diseases, including coronary heart disease, congestive heart failure, stroke, and CKD.^3^, ^4^, ^5^, ^6^, ^7^ In individuals with CKD or diabetes, smoking’s adverse cardiovascular effects are amplified, accelerating kidney dysfunction and increasing all-cause mortality.^8^ The growing prevalence of E-cigarette use and vaping products presents new concerns, because accumulating evidence demonstrates their ability to induce oxidative stress, altered metabolism, vascular stiffness, and thrombosis, predisposing individuals to negative impacts on cardiometabolic health, although long-term data remain limited.^9^ Despite these established links, few studies have examined cigarette smoking and vaping in the context of clusters of conditions that define CKMS stages.^10^

Similarly, patterns of heavy alcohol consumption represent another underexplored yet potentially important modifiable risk behavior in the CKMS population. Epidemiologic and clinical evidence supports the association between excessive alcohol use and increased cardiac complications, including heart failure, arrhythmias, and hypertension, although there remains a paucity of information about the frequency of heavy alcohol consumption among people with CKMS.^11^^,^^12^ Concurrently, meta-analyses suggest a modest but clinically relevant increase in CKD incidence associated with alcohol consumption—particularly at higher intake—due to adverse effects on renal function, increasing the risk for albuminuria, proteinuria, and reduced glomerular filtration rate, likely mediated by oxidative stress and inflammatory cytokine activation.^13^ Alcohol’s contribution to metabolic dysfunction is also well documented, as evidenced by associations with components of metabolic syndrome, including hyperlipidemia, hypertriglyceridemia, high fasting glucose, and low high-density lipoprotein cholesterol levels, compounding risks in people with diabetes—a major driver of CKMS.^14^^,^^15^ However, similar to cigarette smoking and vaping, CKMS stage–specific odds of heavy alcohol consumption, especially across sociodemographic groups, remain less visited in the literature.

Given these gaps, this study aims to assess the odds of current cigarette smoking, current E-cigarette smoking or vaping, and heavy drinking (alcohol consumption) by CKMS stages by sex and age groups. Acknowledging the established associations of these health risk behaviors with several individual components of CKMS, the broad objective of this study is to explore whether the odds vary across different CKMS stages. The secondary aim is to assess whether the probability of health risk behaviors varies by sex at different stages of CKMS. This exploratory research is thus intended to facilitate the understanding of certain modifiable health-risk behaviors among individuals across CKMS stages and may help to comprehend stage-specific behavioral strategies to be integrated within CKMS management.

## METHODS

### Study Population

This cross-sectional study used publicly available data from the Behavioral Risk Factor Surveillance System (BRFSS) survey, an annual telephone-based survey of U.S. residents aged ≥18 years. The BRFSS collects information about various health conditions and health behaviors. The authors pooled cross-sectional data from the latest waves (i.e., 2021 and 2023) of the BRFSS survey, which contained necessary information about conditions for determining CKMS stages. Observations with missing data were excluded from the analysis (318,622 of 856,745), and the analytical sample size was 528,123. It should be noted that 2 key components of CKMS, namely hypertension and high cholesterol, were not available in the 2022 and 2024 BRFSS. As such, data from those 2 waves were not included. Publicly available anonymized data were used, which met the NIH definition of exempt human subjects research (Exemption 4). The study was determined to be “Not Human Subjects Research” by the Augusta University IRB as defined under federal regulations (45 CFR 46.102). Therefore, IRB review was not required.

### Measures

To determine the CKMS stages, self-reported data on heart attack (myocardial infarction), coronary heart disease, stroke, prediabetes, diabetes, prehypertension, hypertension, high cholesterol, CKD, and overweight/obesity (i.e., BMI ≥25 kg/m^2^) were used. Stages 2 and 3 were consolidated into 1 category because data on subclinical CVD, predicted CVD risk, and risk classification of CKD were not available.

Having a BMI <25 kg/m^2^ and not having prediabetes, diabetes, hypertension, prehypertension, high cholesterol, CKD, and CVD constitutes CKMS Stage 0. Having a BMI ≥25 kg/m^2^ or having prediabetes and not having diabetes, hypertension, high cholesterol, CKD, and CVD constitute CKMS Stage 1. Not having CVD and having any of the following conditions: high cholesterol, hypertension, prediabetes, diabetes, metabolic syndrome (i.e., having at least 3 of the following conditions: BMI ≥25 kg/m^2^, diabetes, hypertension, and high cholesterol), or CKD constitute CKMS Stages 2/3. Finally, having CVD and any of the following conditions: BMI ≥25 kg/m^2^, prediabetes, diabetes, hypertension, high cholesterol, metabolic syndrome, or CKD constitutes CKMS Stage 4. A detailed description of using BRFSS data for determining CKMS stages is available elsewhere.^16^ The exposure variable is a categorical variable indicating different stages of CKMS.

Outcome variables are all binary variables indicating health risk behaviors. The first outcome, current smoking, indicates whether a respondent smoked at least 100 cigarettes in their entire life and currently smokes cigarettes every day or on some days. The second outcome, current vaping, indicates whether the respondent currently uses E-cigarettes or other vaping products every day or on some days. The last outcome, heavy drinking, indicates having more than 14 drinks per week for males and 7 drinks per week for females.

### Statistical Analysis

First, the prevalence of current smoking, current vaping, and heavy drinking was examined by CKMS stage for all adults and by demographic subgroups (i.e., male, female, ages 18–49 years, ages 50–64 years, and ages ≥65 years). Next, separate multivariable binomial logistic regression models were estimated to obtain predictive margins of health risk behaviors for CKMS, as follows:

$$\text{logit}\left(Y_{i}^{k}\right)=\sum_{j=1}^{3}\gamma_{j}\text{CKM}S_{j,i}+\beta_{1}\text{Femal}e_{i}+\beta_{2}X_{i}+\text{Yea}r_{y}+\text{Mont}h_{m}+\text{Stat}e_{s}$$

Here, Y^k^ refers to k^th^ outcome (e.g., current smoking, vaping, or heavy drinking), CKMS_j_ refers to j^th^ stage, and ***X*** is a vector of covariates including age, biological sex (male and female), race and ethnicity (non-Hispanic White, non-Hispanic Black, non-Hispanic Asian, Hispanic, and non-Hispanic other), marital status (married, widowed/divorced/separated, never married, and other), educational attainment (high school or less, some college, and college graduate), household income (low, medium, and high), and employment status (employed, retired, homemaker/student, and other). Year_y_ is the survey year-fixed effect, Month_m_ is the interview month-fixed effect, and State_s_ is the state of residence–fixed effect. Models were estimated for all observations and for sex and age subgroups.

Predictive margins of respective health-risk behaviors at different CKMS stages were estimated. Next, to assess sex differences, a model with an interaction of biological sex and CKMS stages was estimated as follows:

$$\text{logit}\left(Y_{i}^{k}\right)=\sum_{j=1}^{3}\rho_{j}\text{CKM}S_{j,i}+\alpha_{1}\text{Femal}e_{i}+\sum_{j=1}^{3}\delta_{j}\left(\text{Femal}e_{i}\times \;\text{CKM}S_{j,1}\right)+\alpha_{2}X_{i}+\text{Yea}r_{y}+\text{Mont}h_{m}+\text{Stat}e_{s}$$

Predictive margins for males and females were calculated at different CKMS stages. Statistically significant differences in predictive margins between females and males (i.e., average marginal effects of female sex) at certain CKMS stages indicate sex differences in the odds of health-risk behavior at that stage. Estimates were obtained using complex survey weights adjusted for the pooled structure of the data, entailing the sampling frame of the BRFSS.^17^ The delta method was used to calculate SEs of predictive margins. Statistical analyses were conducted using Stata 18.0 software.

## RESULTS

In the sample, the prevalence rates of CKMS stages 0, 1, 2/3, and 4 were 16.39%, 26.84%, 47.85%, and 8.92%, respectively. Table 1 shows the prevalence of CKMS in the sample by sex, age categories, and stage. The prevalence of advanced CKMS stage (i.e., Stage 4) increases with age and was higher among men than among women. Descriptive statistics of the study sample, stratified by sociodemographic subgroups and CKMS stages, are presented in Tables A.1–3 (available online). Crude ORs of current smoking, current vaping, and heavy drinking for demographic and socioeconomic attributes are presented in Tables A.4–6 (available online), respectively.

**Table 1:** Prevalence of CKMS Stages

Group	Stage 0	Stage 1	Stage 2/3	Stage 4	*n*
All	16.39	26.84	47.85	8.92	538,119
	(16.14, 16.64)	(26.55, 27.12)	(47.53, 48.18)	(8.75, 9.09)	
Male	13.57	26.68	49.23	10.51	260,430
	(13.24, 13.90)	(26.29, 27.08)	(48.79, 49.68)	(10.26, 10.77)	
Female	19.2	26.99	46.48	7.33	277,689
	(18.83, 19.57)	(26.58, 27.40)	(46.01, 46.94)	(7.10, 7.56)	
Age 18-49	24.37	39.35	33.95	2.34	176,902
	(23.93, 24.81)	(38.87, 39.83)	(33.48, 34.42)	(2.18, 2.49)	
Age 50-64	10.65	20.94	58.21	10.2	157,711
	(10.30, 11.00)	(20.45, 21.42)	(57.62, 58.81)	(9.83, 10.57)	
Age 65+	7.71	9.66	62.56	20.07	203,506
	(7.40, 8.02)	(9.33, 9.98)	(62.01, 63.12)	(19.62, 20.51)	

*Note*: Prevalence rates were estimated using the complex survey weights that entailed the sampling frame of the BRFSS, adjusted for the pooled structure of the data. The 95% CIs are in parentheses.

BRFSS, Behavioral Risk Factor Surveillance System; CKMS, cardiovascular–kidney–metabolic syndrome.

The prevalence of current smoking was 12.14%. Whereas it was 11.02% among those with CKMS Stage 0, it was 12.24% and 17.24% among those with CKMS Stages 2/3 and 4, respectively (Table 2). Except for the ≥65 years age group, a higher prevalence of current smoking among adults with CKMS Stage 4 was evident in all other subgroups.

**Table 2:** Prevalence of Current Smoking, Vaping, and Heavy Drinking by CKMS Stages

		CKMS Stages			
Risk Behaviors by Group	All	Stage 0	Stage 1	Stages 2/3	Stage 4
A. Current smoking					
All	12.14	11.02	10.95	12.24	17.24
	(11.94, 12.35)	(10.52, 11.52)	(10.58, 11.32)	(11.94, 12.54)	(16.52, 17.97)
Male	13.00	14.47	12.06	12.37	16.42
	(12.70, 13.31)	(13.56, 15.38)	(11.49, 12.63)	(11.94, 12.80)	(15.47, 17.38)
Female	11.28	8.59	9.85	12.1	18.42
	(11.02, 11.55)	(8.05, 9.13)	(9.38, 10.33)	(11.69, 12.52)	(17.29, 19.54)
Age 18–49 years	12.30	9.88	11.1	14.4	26.85
	(11.99, 12.61)	(9.27, 10.49)	(10.67, 11.54)	(13.81, 14.98)	(24.01, 29.70)
Age 50–64 years	14.52	14.95	11.65	13.78	24.16
	(14.11, 14.93)	(13.86, 16.04)	(10.77, 12.52)	(13.25, 14.32)	(22.68, 25.63)
Age ≥65 years	9.09	11.64	7.98	8.33	11.02
	(8.76, 9.42)	(10.32, 12.97)	(6.92, 9.03)	(7.91, 8.74)	(10.27, 11.78)
B. Current vaping					
All	6.07	8.46	7.17	4.97	4.28
	(5.90, 6.23)	(7.98, 8.94)	(6.86, 7.47)	(4.74, 5.19)	(3.75, 4.81)
Male	6.72	11.25	7.94	5.37	4.06
	(6.48, 6.95)	(10.42, 12.08)	(7.50, 8.38)	(5.07, 5.68)	(3.29, 4.84)
Female	5.42	6.49	6.4	4.53	4.59
	(5.19, 5.65)	(5.93, 7.06)	(5.97, 6.84)	(4.20, 4.87)	(3.94, 5.23)
Age 18–49 years	10.22	11.03	9.34	10.43	13.63
	(9.91, 10.54)	(10.37, 11.70)	(8.91, 9.77)	(9.84, 11.03)	(10.81, 16.45)
Age 50–64 years	3.34	3.15	2.86	3.17	5.54
	(3.15, 3.54)	(2.68, 3.62)	(2.48, 3.24)	(2.93, 3.40)	(4.48, 6.59)
Age ≥65 years	1.25	1.4	0.98	1.2	1.45
	(1.14, 1.35)	(0.98, 1.82)	(0.73, 1.22)	(1.08, 1.33)	(1.18, 1.72)
C. Heavy drinking					
All	6.21	6.77	6.21	6.4	4.2
	(6.06, 6.37)	(6.35, 7.19)	(5.94, 6.48)	(6.16, 6.64)	(3.81, 4.59)
Male	6.18	5.64	5.98	6.74	4.84
	(5.96, 6.41)	(5.05, 6.23)	(5.60, 6.35)	(6.39, 7.08)	(4.28, 5.40)
Female	6.24	7.57	6.44	6.05	3.28
	(6.02, 6.46)	(6.99, 8.15)	(6.05, 6.83)	(5.72, 6.38)	(2.78, 3.78)
Age 18–49 years	7.05	6.81	6.57	7.78	6.85
	(6.79, 7.31)	(6.26, 7.36)	(6.22, 6.91)	(7.28, 8.29)	(5.32, 8.37)
Age 50–64 years	6.25	7.32	5.63	6.56	4.62
	(5.98, 6.51)	(6.58, 8.05)	(5.14, 6.11)	(6.18, 6.94)	(3.92, 5.31)
Age ≥65 years	4.59	5.67	4.91	4.8	3.38
	(4.33, 4.85)	(4.77, 6.57)	(4.16, 5.66)	(4.45, 5.15)	(2.91, 3.84)

*Note*: Prevalence rates were estimated using the complex survey weights that entailed the sampling frame of the BRFSS, adjusted for the pooled structure of the data. The 95% CIs are in parentheses.

BRFSS, Behavioral Risk Factor Surveillance System; CKMS, cardiovascular–kidney–metabolic syndrome.

Predictive margins of current smoking for CKMS stages, after adjusting for model covariates, are presented in Figure 1. Compared with adults with CKMS Stage 0, the likelihood of current smoking was significantly higher among adults with CKMS Stage 4 and lower among adults with CKMS Stage 1. This pattern was similar for males. Among females, the likelihood of current smoking was higher among adults with CKMS Stages 2/3 and 4. Across age groups, the higher likelihood of current smoking for adults with CKMS Stage 4 was only evident for the 18–49 years subgroup. For adults aged ≥65 years, current smoking was significantly lower among adults with advanced CKMS stages. Figure A.1 (available online) illustrates the comparison between crude and adjusted probabilities of current smoking by CKMS stages, and Table A.7 (available online) presents adjusted ORs for all model covariates.

**Figure 1:**
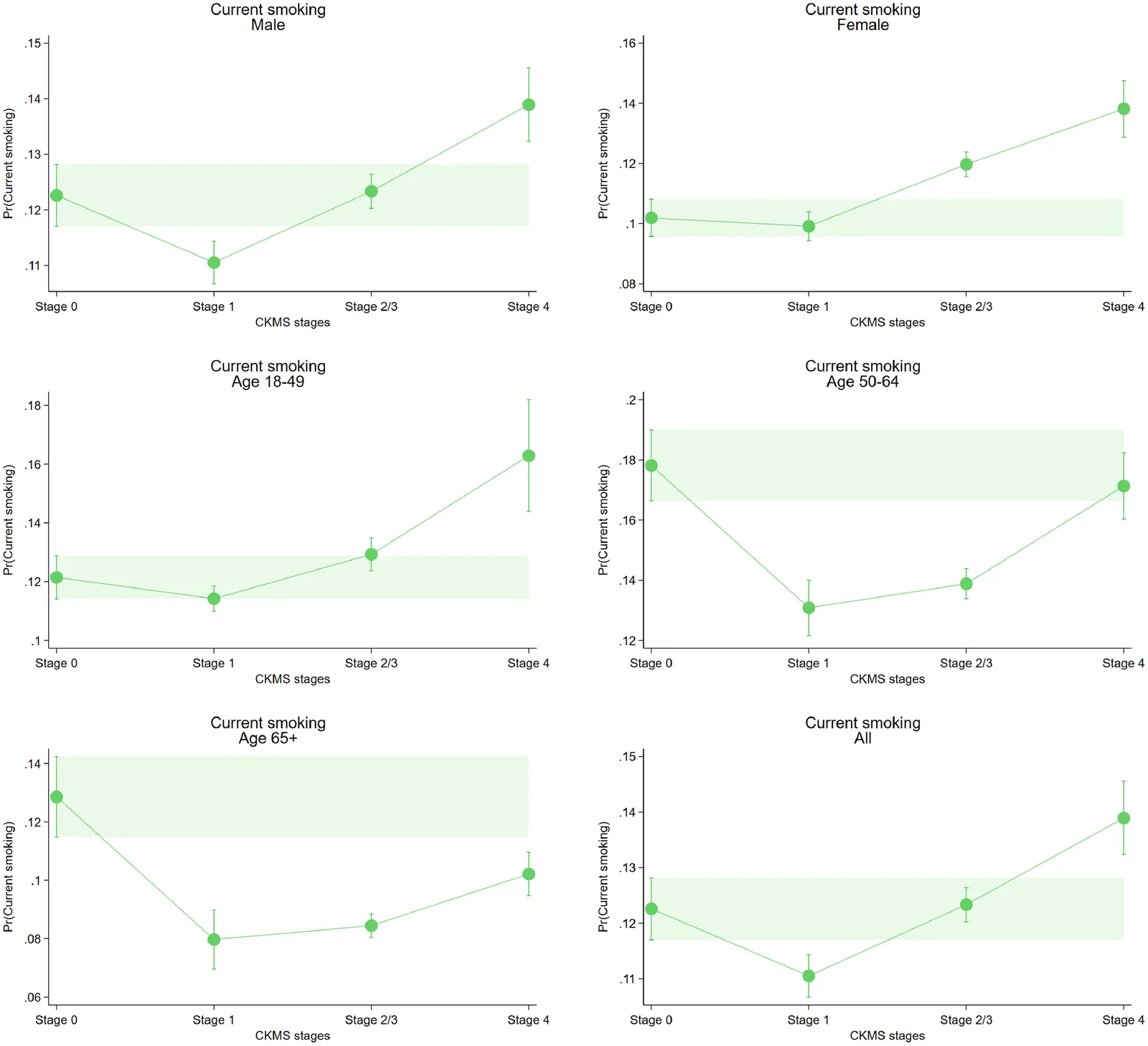
Predictive margins of current smoking by CKMS stages. *Note*: Models were estimated using the complex survey weights that entailed the sampling frame of the BRFSS, adjusted for the pooled structure of the data. Adjustments were made for age, biological sex, race and ethnicity, marital status, educational attainment, income, employment status, survey year-fixed effects, interview month-fixed effects, and state of residence–fixed effects. BRFSS, Behavioral Risk Factor Surveillance System; CKMS, cardiovascular–kidney–metabolic syndrome.

Predictive margins by sex are presented in Figure 2. Although males had a higher probability of current smoking at CKMS Stages 0, 1, and 2/3, no statistically significant difference in the likelihood of current smoking was observed between males and females at CKMS Stage 4. The case was similar for adults aged 18–49 years. For older adults, the likelihood of current smoking differed between males and females only at CKMS Stage 0.

**Figure 2:**
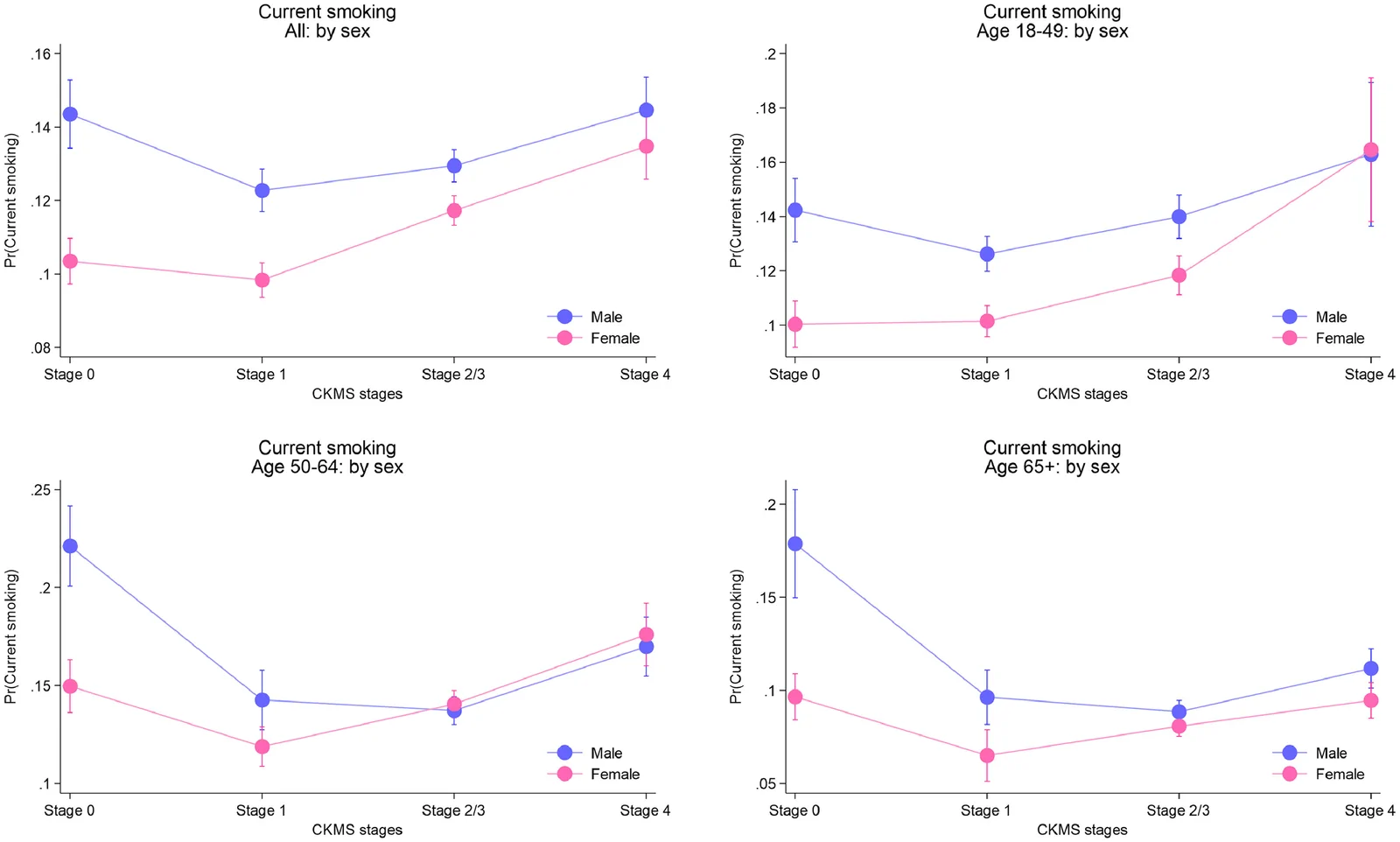
Predictive margins of current smoking by CKMS stages and sex. *Note*: Models were estimated using the complex survey weights that entailed the sampling frame of the BRFSS, adjusted for the pooled structure of the data. Adjustments were made for age, race and ethnicity, marital status, educational attainment, income, employment status, survey year-fixed effects, interview month-fixed effects, and state of residence–fixed effects. CKMS stages and biological sex were interacted in the model to assess the probability by sex. BRFSS, Behavioral Risk Factor Surveillance System; CKMS, cardiovascular–kidney–metabolic syndrome.

Around 6.07% of adults reported current use of E-cigarettes or other vaping products. The prevalence of current vaping was highest (8.46%) among those with CKMS Stage 0 and was relatively lower among those with advanced CKMS stages (Table 2). This pattern was similar in both male and female subgroups. In the 18–49 years and 50–64 years age subgroups, adults with CKMS Stage 4 had the highest prevalence of current vaping. No statistically significant difference in the prevalence of current vaping was observed across CKMS stages in the ≥65 years age subgroup. A comparison between crude and adjusted probabilities of current vaping by CKMS stages is presented in Figure A.2 (available online), and adjusted ORs for model covariates are presented in Table A.8 (available online).

In contrast to crude prevalence rates, the predictive margins of current vaping show a higher probability of vaping among those with CKMS Stage 4 (Figure 3). This pattern was generally similar across all subgroups except for those aged ≥65 years.

**Figure 3:**
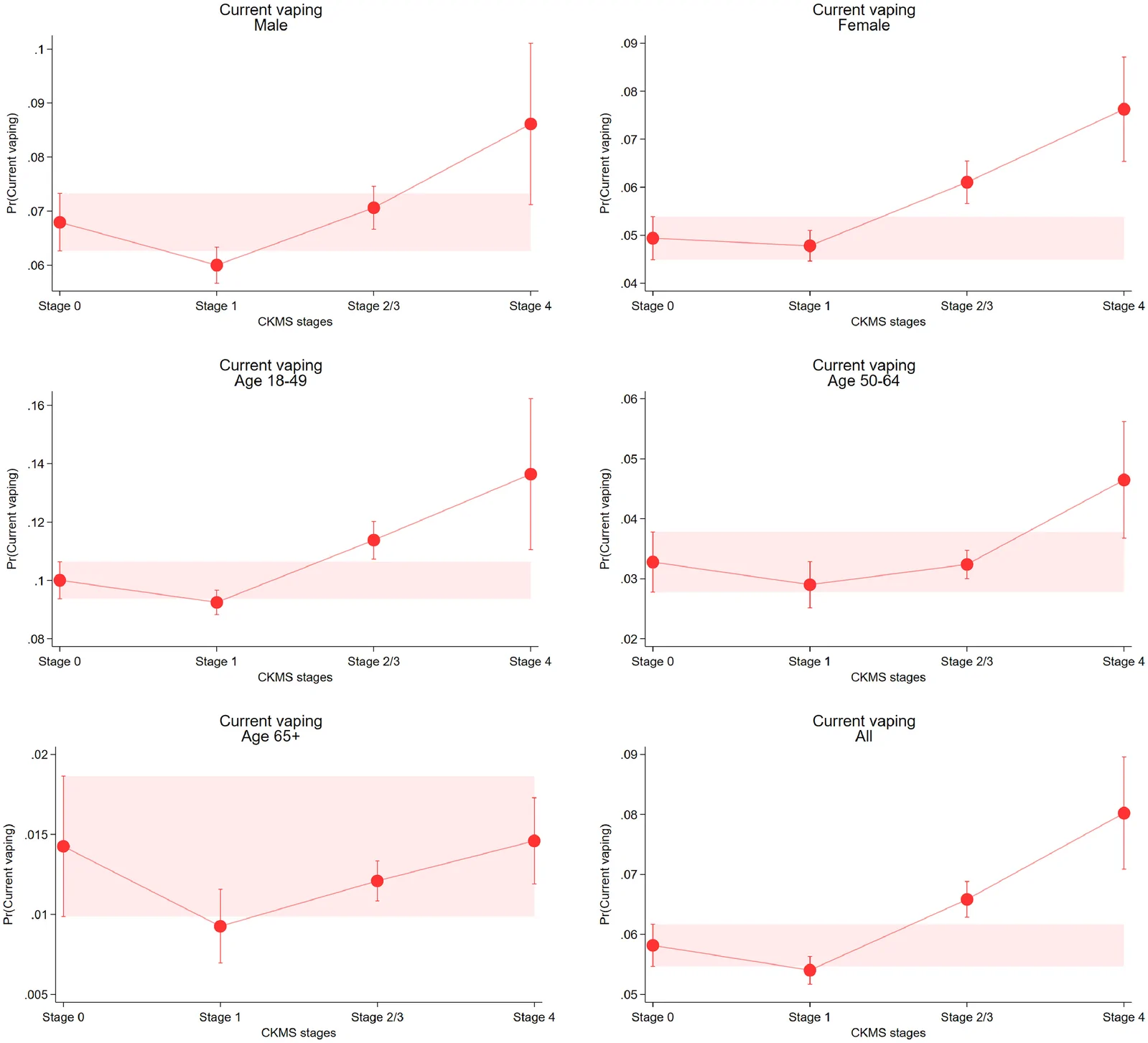
Predictive margins of current vaping by CKMS stages. *Note*: Models were estimated using the complex survey weights that entailed the sampling frame of the BRFSS, adjusted for the pooled structure of the data. Adjustments were made for age, biological sex, race and ethnicity, marital status, educational attainment, income, employment status, survey year-fixed effects, interview month-fixed effects, and state of residence–fixed effects. BRFSS, Behavioral Risk Factor Surveillance System; CKMS, cardiovascular–kidney–metabolic syndrome.

Figure 4 presents the predictive margins of vaping at CKMS stages by sex. Compared with females, males had a higher probability of current vaping at CKMS Stages 0 and 1. However, no differences between males and females were found at CKMS Stages 2/3 and 4. These results were also evident in the 18–49 years age subgroup. In the 50–64 years and ≥65 years subgroups, no differences between males and females were found at any CKMS stage.

**Figure 4:**
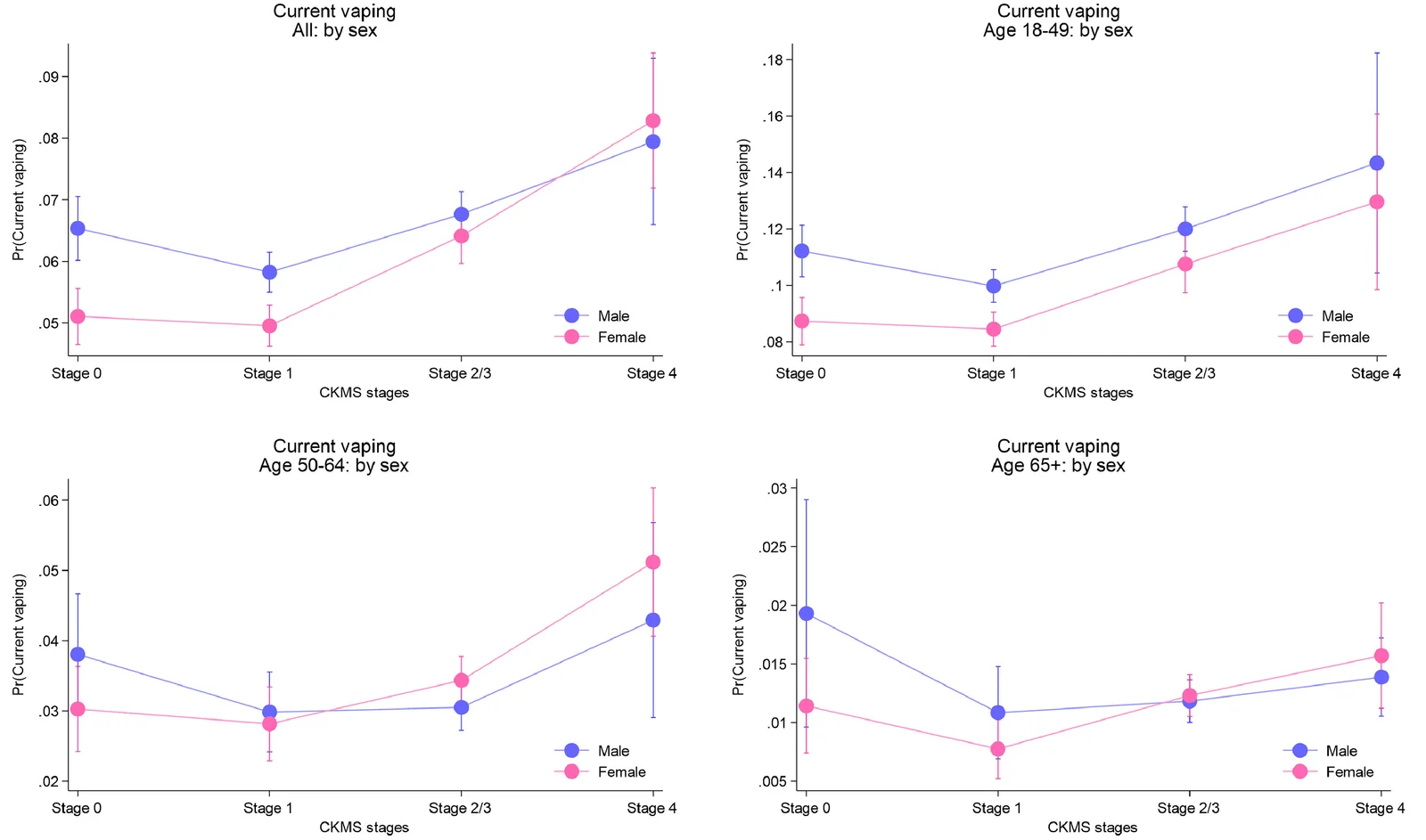
Predictive margins of current vaping by CKMS stages and sex. *Note*: Models were estimated using the complex survey weights that entailed the sampling frame of the BRFSS, adjusted for the pooled structure of the data. Adjustments were made for age, race and ethnicity, marital status, educational attainment, income, employment status, survey year-fixed effects, interview month-fixed effects, and state of residence–fixed effects. CKMS stages and biological sex were interacted in the model to assess the probability by sex. BRFSS, Behavioral Risk Factor Surveillance System; CKMS, cardiovascular–kidney–metabolic syndrome.

The prevalence of heavy drinking was 6.21% in the study sample. It was highest among adults with CKMS Stage 0 (6.77%) and lowest among adults with CKMS Stage 4 (Table 2). Except for the 18–49 years age subgroup, the prevalence of heavy drinking was lowest among adults with CKMS Stage 4 in all subgroups.

Predictive margins also show a lower probability of heavy drinking for adults with CKMS Stage 4 (Figure 5). Although this was observed in the female subsample as well, in the male subsample, there was no difference in the probability of heavy drinking among adults with CKMS Stages 0, 1, and 4. Among males, the predicted probability of heavy drinking was found to be significantly higher for those with CKMS Stage 2/3. No statistically significant difference in the likelihood of heavy drinking by CKMS stages was observed in the 18–49 years age subgroup. In the ≥65 years subgroup, the likelihood of heavy drinking was low for those with CKMS Stage 4, and no differences were observed across CKMS Stages 0, 1, and 2/3. In the 50–64 years subgroup, no specific patterns were observed—the predicted probability was lower for those with CKMS stages 1 and 4 than for those with CKMS Stages 0 and 2/3. The crude and adjusted probabilities of heavy drinking by CKMS stages are illustrated in Figure A.3 (available online), and adjusted ORs for model covariates are presented in Table A.9 (available online).

**Figure 5:**
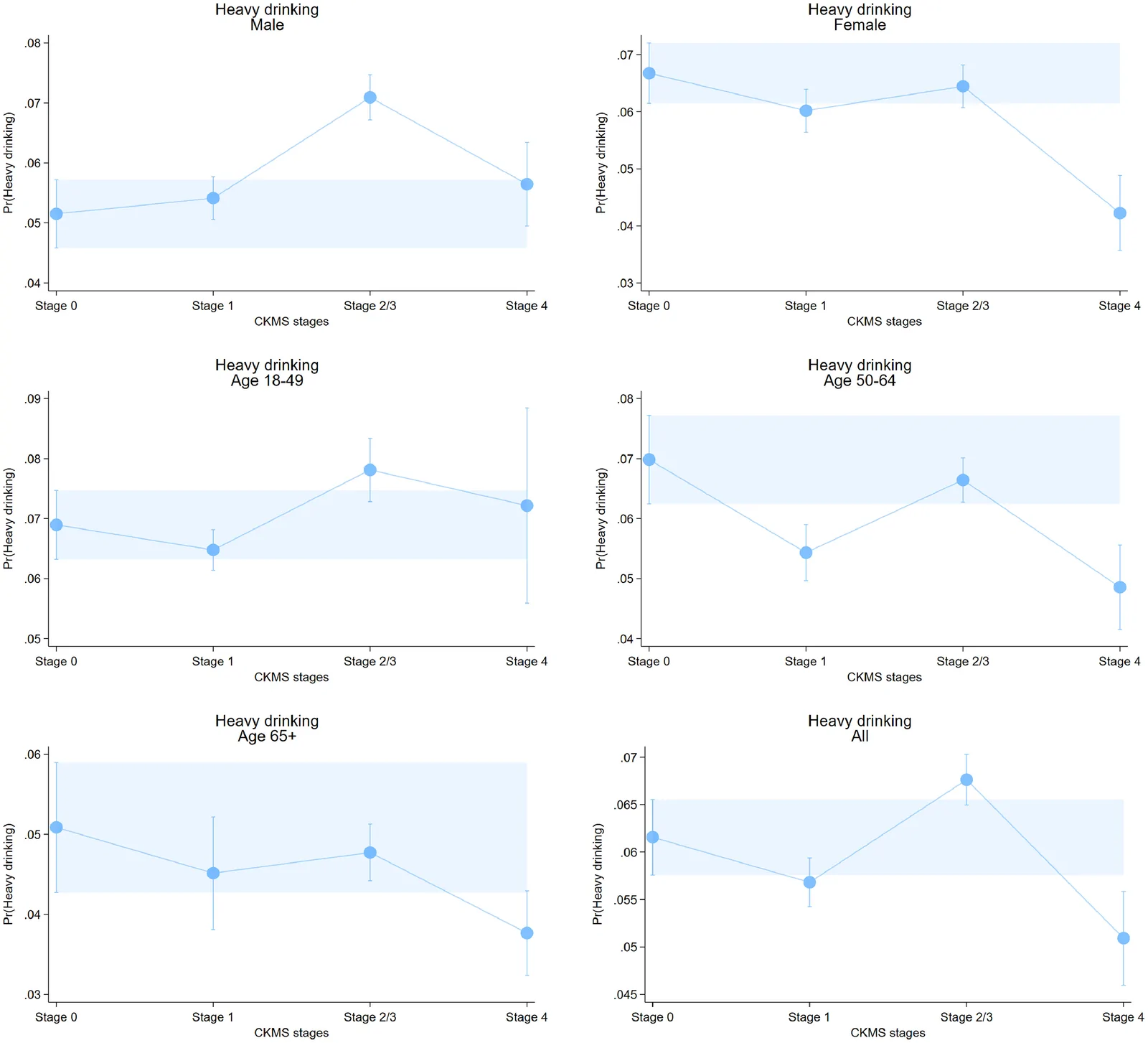
Predictive margins of heavy drinking by CKMS stages. *Note*: Models were estimated using the complex survey weights that entailed the sampling frame of the BRFSS, adjusted for the pooled structure of the data. Adjustments were made for age, biological sex, race and ethnicity, marital status, educational attainment, income, employment status, survey year-fixed effects, interview month-fixed effects, and state of residence–fixed effects. BRFSS, Behavioral Risk Factor Surveillance System; CKMS, cardiovascular–kidney–metabolic syndrome.

Predictive margins at CKMS stages by sex are presented in Figure 6. Whereas men had a higher probability of heavy drinking than women at CKMS Stage 4, women had a higher probability of heavy drinking at CKMS Stages 0 and 1. No statistically significant difference in the likelihood of current heavy drinking was observed between males and females at CKMS Stages 2/3. Across all age groups, no differences between males and females were observed at CKMS Stages 1 and 2/3. In the 18–49 years and ≥65 years age subgroups, females had a higher likelihood of heavy drinking at CKMS Stage 0. In the 50–64 years age subgroup, however, no such difference was observed. Males had a higher likelihood of heavy drinking at CKMS Stage 4 in the 18–49 years age subgroup, and no such differences were observed in the 50–64 years and ≥65 years age subgroups. Average marginal effects of female sex at different CKMS stages are presented in Table A.10 (available online).

**Figure 6:**
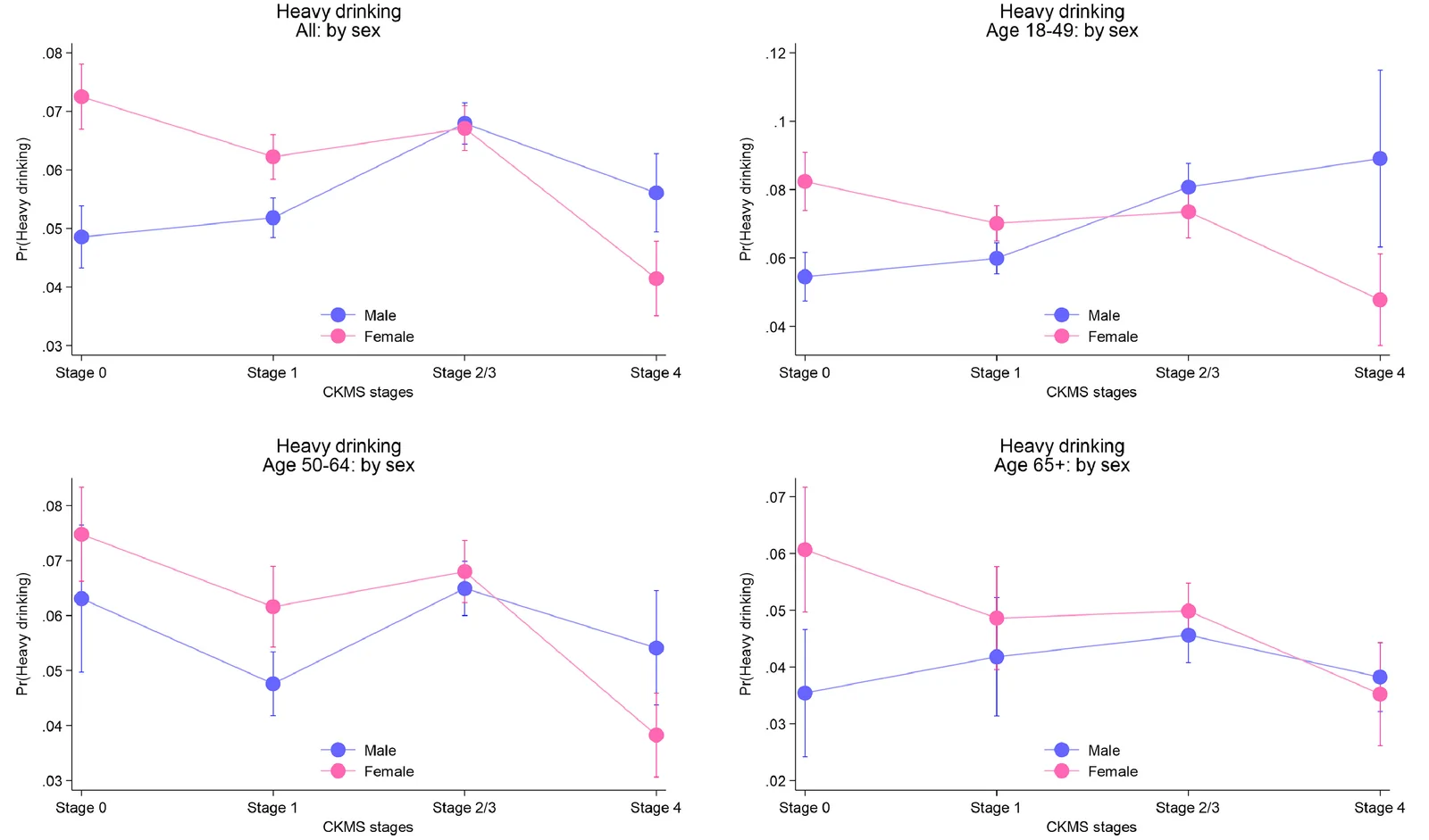
Predictive margins of heavy by CKMS stages and sex. *Note*: Models were estimated using the complex survey weights that entailed the sampling frame of the BRFSS, adjusted for the pooled structure of the data. Adjustments were made for age, race and ethnicity, marital status, educational attainment, income, employment status, survey year-fixed effects, interview month-fixed effects, and state of residence–fixed effects. CKMS stages and biological sex were interacted in the model to assess the probability by sex. BRFSS, Behavioral Risk Factor Surveillance System; CKMS, cardiovascular–kidney–metabolic syndrome.

## DISCUSSION

The results of this nationally representative study indicate that health risk behaviors in the form of smoking, vaping, and heavy drinking vary by CKMS stage, age group, and sex. Among both men and women, the likelihood of current smoking was higher among those at CKMS Stage 4 than among those at lower CKMS stages. This pattern, however, was evident among younger adults (aged 18–49 years) only and not among adults aged 50–64 years and ≥65 years. Current vaping at CKMS Stage 4 was particularly higher among females and adults aged 18–49 years. No difference in the probability of vaping was observed across CKMS stages for adults aged ≥65 years. In contrast, the probability of heavy drinking was lower at CKMS Stage 4 for women and older adults (i.e., aged 50–64 years and ≥65 years). No difference in the probability of heavy drinking was found across CKMS stages for younger adults aged 18–49 years.

Interesting variations were also observed by sex at different CKMS stages. Males had a higher likelihood of current smoking at CKMS Stages 0, 1, and 2/3 than with females; no difference between males and females was observed at CKMS Stage 4. Among older adults aged 50–64 years and ≥65 years, no differences in current smoking between males and females were observed at CKMS Stages 2/3 and 4. For the current vaping outcome, no differences between males and females were observed at CKMS Stages 2/3 and 4, and no differences by CKMS stages were found between males and females aged 50–64 years and ≥65 years. The probability of heavy drinking was higher among females than among males at CKMS Stages 0 and 1. Although no difference between males and females was found at CKMS Stages 2/3, females at CKMS Stage 4 had a lower likelihood of heavy drinking than males at CKMS Stage 4.

These findings highlight the variations in health risk behaviors by CKMS stages, age group, and sex. Owing to data limitations, it was not feasible to investigate the underlying causes of the differential odds at different CKMS stages across age groups and sex. Future research, entailing both quantitative and qualitative approaches, is needed to better understand the underlying causes. Evidence suggests that factors such as heightened psychosocial stress, depression, and anxiety are associated with nicotine dependence and poorer cessation outcomes.^18^ Psychological distress is also a known risk factor for heavy drinking.^19^ Several recent studies have shown an association between depressive symptoms and CKMS^20^, ^21^, ^22^ and a higher risk of anxiety at advanced CKMS stages.^23^ The elevated risk of depression and anxiety at advanced CKMS stages may influence health risk behaviors. Conversely, patients with advanced CKMS stages may continue the same risky health behaviors (e.g., smoking and alcohol drinking), which might contribute to their current health condition.^24^ Future research is needed to disentangle the intertemporal aspects and scientifically investigate the probable pathways.

Women generally report a lower prevalence of smoking, vaping, and heavy drinking than men.^25^, ^26^, ^27^ The study findings suggest that although women were less likely to smoke or vape than men at lower stages of CKMS (e.g., Stage 0 or 1), the gap between males and females did not exist at an advanced CKMS stage (i.e., Stage 4). This highlights an interesting aspect of health risk behavior at an advanced CKMS stage, where commonly observed sex-based differences did not persist. As such, a better understanding of the odds of certain health risk behaviors across CKMS stages, in addition to sex-based differences, may facilitate better management of CKMS conditions.

The study findings have several implications for public health and further research to inform clinical practices. Given the established associations between cigarette smoking and incident heart failure, stroke, and CKD progression,^3^, ^4^, ^5^, ^6^, ^7^, ^8^ persistent tobacco use in advanced CKMS represents a public health concern. Emerging evidence also suggests that ENDS may adversely affect cardiometabolic and vascular health,^9^ further underscoring the importance of addressing vaping within the CKMS framework. Although heavy alcohol consumption was less common in advanced CKMS, particularly among women, men demonstrated a higher likelihood of heavy drinking in intermediate stages (2/3). On the basis of established associations between alcohol use and CVD, CKD, and metabolic dysfunction,^11^, ^12^, ^13^, ^14^, ^15^ continued heavy drinking in these intermediate stages may contribute to progression across the CKMS spectrum.

### Limitations

Although this observational study provides some important insights into health risk behaviors and CKMS, there are some limitations to exploring the association using BRFSS data. First and foremost, owing to data limitations, a clinical diagnosis of CKMS stages was not feasible. Rather, proxy measures from self-reported health conditions were used to construct CKMS stages. Owing to a lack of information about predicted CVD risk and risk classification of CKD, CKMS Stages 2 and 3 were combined into 1 single stage (i.e., Stages 2/3), which may induce some attenuation bias in probability estimates for the combined stage. Information about smoking, vaping, and heavy drinking was self-reported, which could be subject to some social desirability bias.^28^ Furthermore, the respondents were observed at a single point in time, and it was not possible to determine whether health risk behaviors preceded or succeeded CKMS conditions. As such, an exploration of any causal or intertemporal associations was not possible. The estimates should also be interpreted cautiously owing to potential bias from any residual confounding. Despite these limitations, BRFSS is a rich source of information about various health conditions and health behaviors. The use of BRFSS data enabled the authors to adjust for various sociodemographic and socioeconomic attributes, which are important determinants of health behaviors.

## CONCLUSIONS

In this cross-sectional study, higher odds of current smoking and vaping and lower odds of heavy drinking were observed among adults with Stage 4 CKMS than among adults at Stages 0, 1, and 2/3. Some variations in the odds across CKMS stages were also observed among sociodemographic groups. These findings can be used as background knowledge for further research to facilitate stage-, age-, and sex-specific behavioral and clinical interventions to better manage CKMS conditions. Guided by future clinical research findings, CKMS management pathways with embedded tobacco/nicotine cessation and alcohol-reduction interventions may help reduce morbidity, slow disease progression, and improve long-term cardiometabolic outcomes.

## CRediT authorship contribution statement

**Mehek Sharma:** Conceptualization, Investigation, Writing – original draft. **Darshan Chudasama:** Conceptualization, Investigation. **Biplab K. Datta:** Conceptualization, Data curation, Formal analysis, Methodology, Writing – original draft. **Steven S. Coughlin:** Conceptualization, Validation, Writing – review & editing. **Marlo M. Vernon:** Validation, Writing – review & editing. **Jennifer C. Sullivan:** Validation, Writing – review & editing, Funding acquisition, Supervision.

## Appendix Supplementary materials

Image, application 1

## Footnotes

Supplementary material associated with this article can be found in the online version at doi:10.1016/j.focus.2026.100529.
