# Supplementary material for: Cardiovascular–Kidney–Metabolic Syndrome and Health Risk Behaviors in U.S. Adults
Source: AJPM Focus. 2026 Jul 15;5(6):100529. doi: 10.1016/j.focus.2026.100529 (PMC13634515; doi:10.1016/j.focus.2026.100529)
Supplement: Supplementary file 1 [file mmc1.pdf]

Cardiovascular-Kidney-Metabolic Syndrome and Health Risk Behaviors in U.S. Adults

Mehek Sharma BS [1]; Darshan Chudasama BS [1]; Biplab K. Datta PhD [2,3*]; Steven S. Coughlin PhD [2,4]; Marlo M. Vernon PhD [2,3]; Jennifer C. Sullivan PhD [5]

[1]Medical College of Georgia, Augusta University, Augusta, GA, USA. [2]Institute of Public and Preventive Health, School of Public Health, Augusta University, Augusta, GA, USA. [3]Department of Health Management, Economics and Policy, School of Public Health, Augusta University, Augusta, GA, USA. [4]Department of Biostatistics, Data Science and Epidemiology, School of Public Health, Augusta University, Augusta, GA, USA. [5]Department of Physiology, Medical College of Georgia, Augusta University, Augusta, GA, USA.

Journal: **AJPM Focus**

Appendix

Table A.1. Descriptive statistics: average age of study participants.

	All	Male	Female	Age 18-49	Age 50-64	Age 65+
**All**						
Age	50.55	50.27	50.84	35.02	57.10	72.77
	(50.44, 50.66)	(50.12, 50.41)	(50.68, 50.99)	(34.93, 35.11)	(57.05, 57.16)	(72.71, 72.83)
**By CKMS stages**						
Stage 0	41.20	39.00	42.75	32.00	56.58	72.46
	(40.93, 41.46)	(38.59, 39.41)	(42.41, 43.10)	(31.80, 32.19)	(56.43, 56.73)	(72.25, 72.68)
Stage 1	42.59	42.65	42.53	34.52	56.18	71.61
	(42.41, 42.76)	(42.41, 42.89)	(42.28, 42.78)	(34.39, 34.65)	(56.07, 56.30)	(71.44, 71.78)
Stage 2/3	55.59	54.40	56.85	37.45	57.34	72.60
	(55.44, 55.74)	(54.20, 54.60)	(56.62, 57.07)	(37.30, 37.60)	(57.27, 57.41)	(72.52, 72.67)
Stage 4	64.65	64.77	64.48	39.82	58.17	74.00
	(64.36, 64.94)	(64.39, 65.15)	(64.04, 64.92)	(39.26, 40.37)	(58.00, 58.34)	(73.88, 74.13)

Note: Estimates were obtained using complex survey weights entailing the sampling frame of the BRFSS. 95% confidence intervals are in parenthesis.

Table A.2. Descriptive statistics: distribution of study participants.

	Percentage					
	All	Male	Female	Age 18-49	Age 50-64	Age 65+
**Sex**						
Male	49.96	100.00	-	50.32	50.37	48.80
	(49.64, 50.28)	(100.00, 100.00)		(49.82, 50.82)	(49.76, 50.97)	(48.23, 49.38)
Female	50.04	-	100.00	49.68	49.63	51.20
	(49.72, 50.36)		(100.00, 100.00)	(49.18, 50.18)	(49.03, 50.24)	(50.62, 51.77)
**Race & Ethnicity**						
White	61.48	61.93	61.04	52.64	64.48	74.98
	(61.16, 61.81)	(61.47, 62.38)	(60.58, 61.51)	(52.17, 53.12)	(63.86, 65.11)	(74.37, 75.59)
Black	11.81	10.81	12.81	12.52	12.31	9.88
	(11.59, 12.03)	(10.51, 11.11)	(12.49, 13.13)	(12.18, 12.86)	(11.89, 12.73)	(9.51, 10.24)
Hispanic	15.80	15.95	15.64	21.42	13.65	7.47
	(15.50, 16.09)	(15.54, 16.37)	(15.22, 16.06)	(20.97, 21.88)	(13.08, 14.22)	(7.02, 7.93)
Asian	6.23	6.41	6.06	8.06	5.13	4.00
	(6.02, 6.45)	(6.12, 6.69)	(5.74, 6.38)	(7.74, 8.38)	(4.75, 5.51)	(3.58, 4.43)
Other	4.68	4.91	4.45	5.36	4.42	3.67
	(4.55, 4.81)	(4.73, 5.08)	(4.26, 4.64)	(5.14, 5.57)	(4.20, 4.65)	(3.47, 3.87)
**Marital status**						
Married	55.00	57.98	52.03	48.04	64.06	57.89
	(54.68, 55.32)	(57.54, 58.42)	(51.56, 52.49)	(47.55, 48.53)	(63.49, 64.63)	(57.33, 58.45)
WDS	20.19	16.02	24.35	10.55	23.19	35.21
	(19.94, 20.43)	(15.70, 16.34)	(23.98, 24.72)	(10.26, 10.84)	(22.69, 23.68)	(34.66, 35.75)
Never married	19.79	20.95	18.63	33.51	9.63	5.20
	(19.52, 20.06)	(20.57, 21.34)	(18.25, 19.01)	(33.04, 33.99)	(9.29, 9.97)	(4.95, 5.45)
Other	5.02	5.04	5.00	7.89	3.12	1.71
	(4.88, 5.16)	(4.85, 5.24)	(4.80, 5.20)	(7.64, 8.15)	(2.92, 3.32)	(1.55, 1.86)
**Education**						
≤ High school	34.06	36.25	31.89	32.78	34.05	36.55
	(33.75, 34.38)	(35.80, 36.69)	(31.44, 32.33)	(32.29, 33.27)	(33.47, 34.63)	(35.96, 37.13)
Some college	31.15	30.15	32.15	30.49	31.49	32.03
	(30.84, 31.46)	(29.73, 30.58)	(31.71, 32.60)	(30.01, 30.96)	(30.91, 32.08)	(31.50, 32.57)
College degree	34.78	33.60	35.96	36.73	34.45	31.42
	(34.50, 35.07)	(33.22, 33.99)	(35.54, 36.38)	(36.29, 37.17)	(33.90, 35.00)	(30.93, 31.91)
**Income**						
Low income	35.20	31.16	39.22	37.79	30.84	35.27
	(34.88, 35.51)	(30.74, 31.59)	(38.77, 39.68)	(37.30, 38.27)	(30.28, 31.39)	(34.70, 35.84)
Middle income	47.08	49.01	45.16	49.40	47.79	41.82
	(46.76, 47.40)	(48.56, 49.45)	(44.70, 45.62)	(48.91, 49.89)	(47.19, 48.40)	(41.25, 42.39)
High income	17.72	19.83	15.61	12.81	21.37	22.92
	(17.49, 17.95)	(19.49, 20.17)	(15.30, 15.93)	(12.51, 13.12)	(20.88, 21.85)	(22.45, 23.38)
Employment						
Employed	59.36	64.74	53.99	77.05	66.39	17.32
	(59.05, 59.67)	(64.32, 65.15)	(53.54, 54.45)	(76.61, 77.48)	(65.84, 66.94)	(16.87, 17.76)
Retired	21.91	21.51	22.30	0.60	12.44	73.70
	(21.67, 22.14)	(21.19, 21.84)	(21.96, 22.63)	(0.51, 0.70)	(12.08, 12.80)	(73.17, 74.23)
Homemaker/student	7.38	3.05	11.71	11.69	4.21	2.79
	(7.19, 7.58)	(2.88, 3.22)	(11.38, 12.05)	(11.34, 12.04)	(3.93, 4.49)	(2.57, 3.00)
Other	11.35	10.70	12.00	10.66	16.96	6.19
	(11.14, 11.56)	(10.41, 10.99)	(11.70, 12.30)	(10.35, 10.97)	(16.53, 17.39)	(5.86, 6.53)
Observations	538,123	260,431	277,692	176,906	157,711	203,506

Note: Estimates were obtained using complex survey weights entailing the sampling frame of the BRFSS. 95% confidence intervals are in parenthesis. Percentages add to 100 across rows for respective characteristics. WDS refers to widowed/divorced/separated.

Table A.3. Descriptive statistics: distribution of study participants by CKMS stages.

	Percentage			
	CKMS Stage 0	CKMS Stage 1	CKMS Stage 2/3	CKMS Stage 4
**Sex**				
Male	41.37	49.67	51.40	58.88
	(40.55, 42.19)	(49.05, 50.29)	(50.94, 51.86)	(57.86, 59.90)
Female	58.63	50.33	48.60	41.12
	(57.81, 59.45)	(49.71, 50.95)	(48.14, 49.06)	(40.10, 42.14)
**Race & Ethnicity**				
White	61.09	57.20	62.67	68.75
	(60.25, 61.93)	(56.59, 57.81)	(62.20, 63.14)	(67.72, 69.78)
Black	8.40	12.19	12.58	12.81
	(7.89, 8.90)	(11.76, 12.62)	(12.26, 12.90)	(11.99, 13.64)
Hispanic	14.97	20.35	14.67	9.63
	(14.21, 15.72)	(19.78, 20.92)	(14.25, 15.10)	(8.85, 10.41)
Asian	10.89	5.23	5.75	3.27
	(10.24, 11.53)	(4.88, 5.58)	(5.44, 6.07)	(2.76, 3.78)
Other	4.65	5.04	4.33	5.53
	(4.29, 5.02)	(4.77, 5.30)	(4.15, 4.50)	(5.12, 5.94)
**Marital status**				
Married	48.25	53.72	58.40	53.03
	(47.43, 49.07)	(53.10, 54.33)	(57.94, 58.86)	(52.02, 54.04)
WDS	13.24	14.40	23.07	34.91
	(12.72, 13.76)	(13.97, 14.82)	(22.70, 23.44)	(33.96, 35.86)
Never married	32.23	25.11	14.48	9.42
	(31.43, 33.03)	(24.56, 25.67)	(14.13, 14.82)	(8.78, 10.05)
Other	6.28	6.77	4.05	2.64
	(5.89, 6.68)	(6.46, 7.08)	(3.87, 4.23)	(2.34, 2.94)
**Education**				
≤ High school	28.67	33.09	34.68	43.62
	(27.86, 29.48)	(32.49, 33.70)	(34.21, 35.14)	(42.62, 44.61)
Some college	27.67	31.86	31.50	33.57
	(26.87, 28.46)	(31.25, 32.47)	(31.06, 31.94)	(32.58, 34.55)
College degree	43.66	35.05	33.82	22.82
	(42.87, 44.45)	(34.50, 35.59)	(33.41, 34.24)	(22.08, 23.55)
**Income**				
Low income	31.82	35.15	34.08	47.54
	(31.01, 32.64)	(34.55, 35.75)	(33.63, 34.53)	(46.53, 48.54)
Middle income	50.55	48.92	46.88	36.28
	(49.71, 51.39)	(48.30, 49.54)	(46.41, 47.34)	(35.33, 37.24)
High income	17.62	15.93	19.04	16.18
	(17.05, 18.19)	(15.49, 16.37)	(18.71, 19.38)	(15.45, 16.91)
Employment				
Employed	67.60	74.02	54.29	27.32
	(66.82, 68.38)	(73.48, 74.56)	(53.83, 54.74)	(26.36, 28.28)
Retired	10.57	8.32	28.61	47.64
	(10.13, 11.01)	(8.02, 8.62)	(28.23, 29.00)	(46.67, 48.61)
Homemaker/student	13.28	8.85	5.32	3.21
	(12.67, 13.89)	(8.46, 9.23)	(5.08, 5.56)	(2.75, 3.66)
Other	8.55	8.81	11.78	21.83
	(8.06, 9.05)	(8.44, 9.17)	(11.47, 12.08)	(20.96, 22.70)
Observations	76,251	124,207	278,236	59,429

Note: Estimates were obtained using complex survey weights entailing the sampling frame of the BRFSS. 95% confidence intervals are in parenthesis. Percentages add to 100 across rows for respective characteristics. WDS refers to widowed/divorced/separated.

Table A.4. Crude odds ratios of current smoking for demographic and socioeconomic attributes

	All	Male	Female	Age 18-49	Age 50-64	Age 65+
**Demography**						
Age	0.998***	0.995***	1.002*	1.032***	1.007	0.925***
	(0.997, 0.999)	(0.994, 0.997)	(1.000, 1.003)	(1.028, 1.035)	(0.999, 1.015)	(0.918, 0.933)
Female	0.851***	-	-	0.743***	0.976	0.960
	(0.819, 0.884)			(0.701, 0.786)	(0.915, 1.042)	(0.886, 1.040)
**Race & Ethnicity**						
White	Ref.	Ref.	Ref.	Ref.	Ref.	Ref.
Black	1.084**	1.362***	0.865***	0.965	1.026	1.361***
	(1.025, 1.146)	(1.258, 1.473)	(0.799, 0.936)	(0.888, 1.050)	(0.934, 1.128)	(1.206, 1.534)
Hispanic	0.709***	0.961	0.473***	0.684***	0.545***	0.928
	(0.656, 0.767)	(0.867, 1.065)	(0.420, 0.533)	(0.620, 0.756)	(0.466, 0.637)	(0.734, 1.173)
Asian	0.439***	0.589***	0.290***	0.411***	0.317***	0.736
	(0.381, 0.505)	(0.504, 0.688)	(0.218, 0.386)	(0.348, 0.485)	(0.253, 0.398)	(0.452, 1.197)
Other	1.409***	1.539***	1.274***	1.313***	1.385***	1.473***
	(1.304, 1.524)	(1.389, 1.705)	(1.129, 1.437)	(1.170, 1.474)	(1.212, 1.583)	(1.261, 1.722)
**Marital status**						
Married	Ref.	Ref.	Ref.	Ref.	Ref.	Ref.
WDS	2.273***	2.564***	2.151***	2.590***	2.824***	1.936***
	(2.173, 2.377)	(2.398, 2.741)	(2.025, 2.285)	(2.391, 2.805)	(2.622, 3.041)	(1.777, 2.109)
Never married	1.544***	1.600***	1.474***	1.318***	2.219***	1.678***
	(1.466, 1.627)	(1.491, 1.716)	(1.364, 1.593)	(1.230, 1.413)	(2.003, 2.459)	(1.457, 1.933)
Other	1.902***	2.031***	1.778***	1.650***	2.329***	2.194***
	(1.749, 2.068)	(1.820, 2.266)	(1.563, 2.024)	(1.482, 1.837)	(1.994, 2.722)	(1.635, 2.943)
**Education**						
College degree	Ref.	Ref.	Ref.	Ref.	Ref.	Ref.
≤ High school	4.525***	4.490***	4.496***	4.611***	5.386***	3.506***
	(4.319, 4.741)	(4.207, 4.791)	(4.207, 4.805)	(4.294, 4.951)	(4.978, 5.827)	(3.188, 3.856)
Some college	3.098***	2.921***	3.291***	3.136***	3.282***	2.859***
	(2.949, 3.254)	(2.722, 3.134)	(3.073, 3.523)	(2.915, 3.374)	(3.013, 3.574)	(2.577, 3.172)
**Income**						
High income	Ref.	Ref.	Ref.	Ref.	Ref.	Ref.
Low income	2.642***	2.694***	2.756***	2.215***	3.007***	3.119***
	(2.483, 2.810)	(2.476, 2.931)	(2.517, 3.016)	(2.016, 2.435)	(2.708, 3.339)	(2.739, 3.552)
Middle income	1.197***	1.209***	1.210***	1.045	1.127*	1.570***
	(1.124, 1.276)	(1.110, 1.317)	(1.103, 1.328)	(0.948, 1.152)	(1.013, 1.254)	(1.374, 1.793)
**Employment**						
Employed	Ref.	Ref.	Ref.	Ref.	Ref.	Ref.
Retired	0.832***	0.782***	0.914**	1.361	1.263***	1.098
	(0.793, 0.873)	(0.731, 0.838)	(0.854, 0.978)	(0.960, 1.931)	(1.141, 1.399)	(0.984, 1.226)
Homemaker/student	0.710***	0.474***	0.870**	0.613***	1.088	1.013
	(0.647, 0.779)	(0.375, 0.600)	(0.785, 0.966)	(0.544, 0.690)	(0.909, 1.301)	(0.787, 1.304)
Other	2.583***	2.444***	2.832***	2.284***	3.039***	2.797***
	(2.455, 2.717)	(2.277, 2.623)	(2.636, 3.043)	(2.118, 2.463)	(2.819, 3.277)	(2.332, 3.354)

Note: Models were estimated using the complex survey weights entailing the sampling frame of the BRFSS, adjusted for the pooled structure of data. 95% confidence intervals are in parenthesis. *** $p < 0.001$, ** $p < 0.01$, * $p < 0.05$. WDS refers to widowed/divorced/separated.

Table A.5. Crude odds ratios of current vaping for demographic and socioeconomic attributes

	All	Male	Female	Age 18-49	Age 50-64	Age 65+
**Demography**						
Age	0.948***	0.945***	0.952***	0.949***	0.957***	0.924***
	(0.946, 0.950)	(0.942, 0.947)	(0.949, 0.954)	(0.945, 0.953)	(0.945, 0.970)	(0.908, 0.940)
Female	0.796***	-	-	0.739***	1.069	1.047
	(0.751, 0.844)			(0.688, 0.793)	(0.945, 1.209)	(0.884, 1.239)
**Race & Ethnicity**						
White	Ref.	Ref.	Ref.	Ref.	Ref.	Ref.
Black	0.780***	0.927	0.652***	0.639***	0.704***	0.892
	(0.704, 0.864)	(0.801, 1.074)	(0.568, 0.747)	(0.565, 0.722)	(0.573, 0.866)	(0.631, 1.261)
Hispanic	0.967	1.093	0.826*	0.708***	0.472***	0.778
	(0.871, 1.074)	(0.963, 1.240)	(0.690, 0.988)	(0.632, 0.794)	(0.350, 0.636)	(0.474, 1.279)
Asian	0.790*	0.873	0.690*	0.589***	0.561	0.372***
	(0.657, 0.949)	(0.720, 1.058)	(0.483, 0.986)	(0.485, 0.716)	(0.290, 1.086)	(0.210, 0.661)
Other	1.380***	1.363***	1.392***	1.142	0.950	1.468*
	(1.231, 1.546)	(1.168, 1.590)	(1.176, 1.648)	(0.998, 1.306)	(0.756, 1.194)	(1.083, 1.990)
**Marital status**						
Married	Ref.	Ref.	Ref.	Ref.	Ref.	Ref.
WDS	1.391***	1.559***	1.311***	1.951***	1.961***	1.492***
	(1.290, 1.501)	(1.392, 1.747)	(1.184, 1.452)	(1.752, 2.173)	(1.720, 2.236)	(1.252, 1.779)
Never married	3.287***	3.451***	3.086***	2.281***	1.404**	1.051
	(3.064, 3.526)	(3.154, 3.776)	(2.758, 3.453)	(2.104, 2.473)	(1.122, 1.755)	(0.699, 1.580)
Other	3.321***	3.646***	2.995***	2.402***	1.943***	1.401
	(3.001, 3.675)	(3.205, 4.148)	(2.546, 3.523)	(2.145, 2.690)	(1.446, 2.613)	(0.912, 2.154)
**Education**						
College degree	Ref.	Ref.	Ref.	Ref.	Ref.	Ref.
≤ High school	2.330***	2.177***	2.476***	2.690***	2.274***	1.688***
	(2.175, 2.496)	(1.989, 2.381)	(2.224, 2.756)	(2.478, 2.921)	(1.970, 2.624)	(1.359, 2.097)
Some college	2.096***	1.956***	2.272***	2.307***	2.272***	1.537***
	(1.958, 2.243)	(1.788, 2.141)	(2.050, 2.518)	(2.128, 2.500)	(1.954, 2.643)	(1.240, 1.905)
**Income**						
High income	Ref.	Ref.	Ref.	Ref.	Ref.	Ref.
Low income	1.722***	1.512***	2.242***	1.273***	1.619***	1.697***
	(1.581, 1.876)	(1.352, 1.691)	(1.972, 2.548)	(1.145, 1.415)	(1.365, 1.920)	(1.327, 2.170)
Middle income	1.267***	1.198**	1.459***	0.926	1.179	1.331*
	(1.163, 1.380)	(1.073, 1.338)	(1.278, 1.666)	(0.833, 1.029)	(0.985, 1.412)	(1.041, 1.702)
**Employment**						
Employed	Ref.	Ref.	Ref.	Ref.	Ref.	Ref.
Retired	0.211***	0.190***	0.244***	1.329	0.965	0.930
	(0.189, 0.236)	(0.166, 0.217)	(0.205, 0.291)	(0.664, 2.660)	(0.809, 1.152)	(0.761, 1.136)
Homemaker/student	1.060	1.538***	1.040	0.930	0.972	0.729
	(0.956, 1.174)	(1.288, 1.836)	(0.912, 1.187)	(0.833, 1.038)	(0.684, 1.383)	(0.403, 1.321)
Other	1.197***	1.114	1.338***	1.365***	1.875***	1.856***
	(1.107, 1.293)	(0.989, 1.255)	(1.207, 1.483)	(1.234, 1.509)	(1.642, 2.141)	(1.327, 2.594)

Note: Models were estimated using the complex survey weights entailing the sampling frame of the BRFSS, adjusted for the pooled structure of data. 95% confidence intervals are in parenthesis. *** $p < 0.001$, ** $p < 0.01$, * $p < 0.05$. WDS refers to widowed/divorced/separated.

Table A.6. Crude odds ratios of heavy drinking for demographic and socioeconomic attributes

	All	Male	Female	Age 18-49	Age 50-64	Age 65+
**Demography**						
Age	0.991***	0.991***	1.003	0.996	0.948***	0.991***
	(0.988, 0.993)	(0.989, 0.993)	(0.999, 1.008)	(0.985, 1.007)	(0.936, 0.959)	(0.988, 0.993)
Female	1.010	-	-	0.975	1.045	1.085
	(0.957, 1.066)			(0.901, 1.055)	(0.955, 1.144)	(0.963, 1.221)
**Race & Ethnicity**						
White	Ref.	Ref.	Ref.	Ref.	Ref.	Ref.
Black	0.650***	0.676***	0.626***	0.749***	0.477***	0.445***
	(0.588, 0.718)	(0.585, 0.782)	(0.545, 0.718)	(0.660, 0.851)	(0.384, 0.593)	(0.355, 0.557)
Hispanic	0.660***	0.765***	0.563***	0.656***	0.449***	0.608
	(0.585, 0.745)	(0.656, 0.893)	(0.463, 0.684)	(0.567, 0.760)	(0.361, 0.558)	(0.354, 1.043)
Asian	0.364***	0.375***	0.354***	0.339***	0.263***	0.499*
	(0.303, 0.438)	(0.288, 0.488)	(0.274, 0.458)	(0.274, 0.419)	(0.187, 0.370)	(0.255, 0.977)
Other	0.862**	0.942	0.783**	0.841*	0.787*	0.771
	(0.772, 0.962)	(0.813, 1.092)	(0.663, 0.923)	(0.732, 0.966)	(0.629, 0.985)	(0.560, 1.062)
**Marital status**						
Married	Ref.	Ref.	Ref.	Ref.	Ref.	Ref.
WDS	1.035	1.272***	0.878**	1.347***	1.088	0.914
	(0.967, 1.107)	(1.154, 1.402)	(0.799, 0.964)	(1.195, 1.520)	(0.981, 1.208)	(0.801, 1.044)
Never married	1.152***	1.125*	1.181**	1.121*	0.809**	0.991
	(1.072, 1.238)	(1.023, 1.238)	(1.061, 1.315)	(1.024, 1.228)	(0.703, 0.932)	(0.767, 1.280)
Other	1.403***	1.449***	1.355***	1.236**	1.511***	1.768***
	(1.254, 1.570)	(1.233, 1.704)	(1.160, 1.584)	(1.072, 1.426)	(1.212, 1.884)	(1.273, 2.457)
**Education**						
College degree	Ref.	Ref.	Ref.	Ref.	Ref.	Ref.
≤ High school	1.003	1.525***	0.646***	1.092	1.114*	0.734***
	(0.941, 1.070)	(1.397, 1.665)	(0.585, 0.713)	(0.995, 1.198)	(1.003, 1.237)	(0.627, 0.860)
Some college	1.077*	1.302***	0.934	1.162**	1.087	0.919
	(1.014, 1.144)	(1.194, 1.420)	(0.858, 1.016)	(1.062, 1.272)	(0.981, 1.204)	(0.817, 1.035)
**Income**						
High income	Ref.	Ref.	Ref.	Ref.	Ref.	Ref.
Low income	0.606***	0.794***	0.463***	0.597***	0.579***	0.539***
	(0.562, 0.654)	(0.713, 0.884)	(0.416, 0.515)	(0.532, 0.669)	(0.510, 0.657)	(0.450, 0.645)
Middle income	0.818***	0.912*	0.724***	0.807***	0.767***	0.717***
	(0.768, 0.871)	(0.833, 0.998)	(0.663, 0.791)	(0.729, 0.894)	(0.691, 0.853)	(0.637, 0.808)
**Employment**						
Employed	Ref.	Ref.	Ref.	Ref.	Ref.	Ref.
Retired	0.689***	0.662***	0.706***	0.940	1.256**	0.755***
	(0.646, 0.734)	(0.607, 0.723)	(0.644, 0.775)	(0.532, 1.660)	(1.089, 1.450)	(0.643, 0.886)
Homemaker/student	0.565***	0.454***	0.572***	0.545***	0.617***	0.509**
	(0.500, 0.639)	(0.357, 0.578)	(0.497, 0.659)	(0.471, 0.631)	(0.485, 0.785)	(0.327, 0.791)
Other	0.728***	0.899	0.584***	0.767***	0.745***	0.750
	(0.657, 0.808)	(0.782, 1.033)	(0.501, 0.681)	(0.669, 0.878)	(0.644, 0.862)	(0.463, 1.217)

Note: Models were estimated using the complex survey weights entailing the sampling frame of the BRFSS, adjusted for the pooled structure of data. 95% confidence intervals are in parenthesis. *** $p < 0.001$, ** $p < 0.01$, * $p < 0.05$. WDS refers to widowed/divorced/separated.

Table A.7. Adjusted odds ratios of current smoking

	All	Male	Female	Age 18-49	Age 50-64	Age 65+
**CKMS stages**						
Stage 0	Ref.	Ref.	Ref.	Ref.	Ref.	Ref.
Stage 1	0.882***	0.809***	0.969	0.926	0.667***	0.563***
	(0.825, 0.943)	(0.734, 0.893)	(0.886, 1.060)	(0.848, 1.011)	(0.589, 0.757)	(0.464, 0.683)
Stage 2/3	1.009	0.857**	1.220***	1.082	0.720***	0.605***
	(0.945, 1.079)	(0.776, 0.947)	(1.119, 1.330)	(0.982, 1.192)	(0.650, 0.796)	(0.524, 0.699)
Stage 4	1.174***	0.987	1.466***	1.472***	0.949	0.760***
	(1.077, 1.281)	(0.871, 1.118)	(1.302, 1.651)	(1.234, 1.757)	(0.834, 1.079)	(0.648, 0.893)
**Demography**						
Age	0.998*	0.998	0.997*	1.043***	0.988**	0.909***
	(0.996, 0.999)	(0.995, 1.000)	(0.995, 0.999)	(1.038, 1.047)	(0.979, 0.996)	(0.902, 0.917)
Female	0.824***			0.769***	0.928*	0.805***
	(0.792, 0.859)			(0.723, 0.819)	(0.865, 0.995)	(0.740, 0.876)
**Race & Ethnicity**						
White	Ref.	Ref.	Ref.	Ref.	Ref.	Ref.
Black	0.782***	0.972	0.614***	0.658***	0.728***	0.948
	(0.735, 0.832)	(0.891, 1.060)	(0.562, 0.671)	(0.599, 0.723)	(0.657, 0.808)	(0.829, 1.085)
Hispanic	0.506***	0.671***	0.340***	0.509***	0.400***	0.597***
	(0.463, 0.552)	(0.597, 0.753)	(0.297, 0.389)	(0.455, 0.571)	(0.335, 0.478)	(0.464, 0.769)
Asian	0.616***	0.810*	0.428***	0.644***	0.487***	0.848
	(0.533, 0.712)	(0.687, 0.956)	(0.321, 0.571)	(0.540, 0.768)	(0.384, 0.618)	(0.520, 1.383)
Other	1.146**	1.272***	1.017	1.098	1.133	1.138
	(1.054, 1.247)	(1.140, 1.420)	(0.892, 1.159)	(0.970, 1.243)	(0.973, 1.320)	(0.965, 1.342)
**Marital status**						
Married	Ref.	Ref.	Ref.	Ref.	Ref.	Ref.
WDS	1.783***	2.021***	1.596***	1.843***	2.097***	1.874***
	(1.697, 1.874)	(1.880, 2.172)	(1.493, 1.707)	(1.687, 2.013)	(1.933, 2.274)	(1.710, 2.054)
Never married	1.212***	1.235***	1.223***	1.561***	1.695***	1.345***
	(1.140, 1.288)	(1.134, 1.344)	(1.120, 1.337)	(1.432, 1.702)	(1.523, 1.886)	(1.149, 1.575)
Other	1.630***	1.654***	1.625***	1.825***	2.095***	2.000***
	(1.488, 1.785)	(1.466, 1.867)	(1.416, 1.864)	(1.621, 2.055)	(1.775, 2.473)	(1.478, 2.706)
**Education**						
College degree	Ref.	Ref.	Ref.	Ref.	Ref.	Ref.
≤ High school	3.426***	3.347***	3.444***	4.003***	3.872***	2.620***
	(3.251, 3.610)	(3.112, 3.600)	(3.192, 3.715)	(3.693, 4.340)	(3.546, 4.228)	(2.350, 2.920)
Some college	2.621***	2.498***	2.739***	2.799***	2.672***	2.353***
	(2.489, 2.760)	(2.319, 2.690)	(2.548, 2.944)	(2.588, 3.026)	(2.447, 2.918)	(2.098, 2.638)
**Income**						
High income	Ref.	Ref.	Ref.	Ref.	Ref.	Ref.
Low income	1.726***	1.758***	1.672***	1.641***	1.729***	1.941***
	(1.610, 1.851)	(1.600, 1.932)	(1.512, 1.849)	(1.472, 1.829)	(1.530, 1.954)	(1.677, 2.246)
Middle income	1.106**	1.128**	1.071	1.007	1.120*	1.318***
	(1.036, 1.181)	(1.033, 1.232)	(0.974, 1.178)	(0.908, 1.117)	(1.004, 1.250)	(1.148, 1.514)
**Employment**						
Employed	Ref.	Ref.	Ref.	Ref.	Ref.	Ref.
Retired	0.619***	0.664***	0.591***	0.866	1.045	1.196**
	(0.580, 0.662)	(0.604, 0.730)	(0.540, 0.648)	(0.600, 1.250)	(0.934, 1.170)	(1.065, 1.344)
Homemaker/student	0.592***	0.340***	0.694***	0.665***	1.022	1.091
	(0.538, 0.652)	(0.267, 0.435)	(0.624, 0.772)	(0.587, 0.752)	(0.847, 1.234)	(0.841, 1.415)
Other	1.497***	1.404***	1.605***	1.374***	1.540***	1.662***
	(1.414, 1.585)	(1.294, 1.523)	(1.482, 1.738)	(1.260, 1.497)	(1.408, 1.684)	(1.366, 2.022)
Constant	0.068***	0.072***	0.054***	0.012***	0.132***	41.471***
	(0.057, 0.080)	(0.057, 0.091)	(0.043, 0.068)	(0.009, 0.017)	(0.074, 0.233)	(21.280, 80.820)
Year Fixed Effect	Y	Y	Y	Y	Y	Y
Month Fixed Effect	Y	Y	Y	Y	Y	Y
State Fixed Effect	Y	Y	Y	Y	Y	Y

Observations	535,632	259,165	276,467	176,238	157,106	202,288

Note: Models were estimated using the complex survey weights entailing the sampling frame of the BRFSS, adjusted for the pooled structure of data. 95% confidence intervals are in parenthesis. *** $p < 0.001$, ** $p < 0.01$, * $p < 0.05$. WDS refers to widowed/divorced/separated.

Table A.8. Adjusted odds ratios of current vaping

	All	Male	Female	Age 18-49	Age 50-64	Age 65+
**CKMS stages**						
Stage 0	Ref.	Ref.	Ref.	Ref.	Ref.	Ref.
Stage 1	0.920	0.867*	0.964	0.913*	0.879	0.644*
	(0.846, 1.000)	(0.776, 0.967)	(0.849, 1.095)	(0.833, 1.000)	(0.714, 1.083)	(0.433, 0.957)
Stage 2/3	1.154**	1.046	1.275***	1.166**	0.987	0.845
	(1.052, 1.266)	(0.930, 1.177)	(1.104, 1.473)	(1.048, 1.297)	(0.826, 1.181)	(0.607, 1.176)
Stage 4	1.463***	1.327*	1.666***	1.466**	1.449*	1.021
	(1.242, 1.722)	(1.047, 1.681)	(1.348, 2.059)	(1.141, 1.883)	(1.089, 1.928)	(0.702, 1.484)
**Demography**						
Age	0.944***	0.942***	0.945***	0.953***	0.941***	0.912***
	(0.941, 0.946)	(0.938, 0.946)	(0.941, 0.949)	(0.948, 0.958)	(0.929, 0.954)	(0.895, 0.929)
Female	0.869***			0.824***	1.069	0.978
	(0.817, 0.925)			(0.764, 0.889)	(0.947, 1.206)	(0.815, 1.174)
**Race & Ethnicity**						
White	Ref.	Ref.	Ref.	Ref.	Ref.	Ref.
Black	0.530***	0.661***	0.414***	0.503***	0.558***	0.704
	(0.476, 0.591)	(0.568, 0.769)	(0.356, 0.482)	(0.443, 0.571)	(0.449, 0.694)	(0.479, 1.034)
Hispanic	0.520***	0.629***	0.408***	0.534***	0.377***	0.578*
	(0.463, 0.584)	(0.547, 0.722)	(0.334, 0.498)	(0.471, 0.605)	(0.274, 0.520)	(0.335, 0.997)
Asian	0.654***	0.727**	0.572***	0.668***	0.671	0.388**
	(0.545, 0.786)	(0.588, 0.899)	(0.416, 0.785)	(0.554, 0.805)	(0.335, 1.344)	(0.211, 0.713)
Other	0.915	0.963	0.857	0.910	0.794	1.192
	(0.811, 1.033)	(0.816, 1.136)	(0.720, 1.019)	(0.790, 1.047)	(0.623, 1.012)	(0.862, 1.650)
**Marital status**						
Married	Ref.	Ref.	Ref.	Ref.	Ref.	Ref.
WDS	1.742***	1.803***	1.644***	1.803***	1.732***	1.554***
	(1.609, 1.886)	(1.603, 2.027)	(1.476, 1.832)	(1.619, 2.009)	(1.505, 1.994)	(1.301, 1.855)
Never married	1.345***	1.315***	1.383***	1.470***	1.297*	0.956
	(1.236, 1.463)	(1.176, 1.471)	(1.217, 1.571)	(1.335, 1.617)	(1.029, 1.636)	(0.620, 1.474)
Other	1.646***	1.731***	1.539***	1.728***	1.898***	1.311
	(1.476, 1.836)	(1.508, 1.988)	(1.293, 1.831)	(1.532, 1.949)	(1.405, 2.564)	(0.853, 2.014)
**Education**						
College degree	Ref.	Ref.	Ref.	Ref.	Ref.	Ref.
≤ High school	2.127***	1.829***	2.496***	2.243***	2.084***	1.473**
	(1.964, 2.303)	(1.648, 2.030)	(2.215, 2.814)	(2.041, 2.465)	(1.766, 2.461)	(1.154, 1.881)
Some college	2.020***	1.810***	2.260***	2.084***	2.103***	1.331*
	(1.877, 2.175)	(1.639, 1.999)	(2.026, 2.522)	(1.913, 2.270)	(1.777, 2.489)	(1.070, 1.656)
**Income**						
High income	Ref.	Ref.	Ref.	Ref.	Ref.	Ref.
Low income	1.120*	1.025	1.259**	1.082	1.111	1.335*
	(1.015, 1.235)	(0.899, 1.168)	(1.089, 1.455)	(0.960, 1.219)	(0.904, 1.367)	(1.010, 1.766)
Middle income	1.035	0.999	1.117	0.980	1.145	1.248
	(0.949, 1.130)	(0.893, 1.118)	(0.974, 1.281)	(0.881, 1.091)	(0.957, 1.371)	(0.968, 1.608)
**Employment**						
Employed	Ref.	Ref.	Ref.	Ref.	Ref.	Ref.
Retired	0.820**	0.832*	0.808	1.542	1.055	1.079
	(0.712, 0.945)	(0.703, 0.986)	(0.642, 1.017)	(0.746, 3.185)	(0.882, 1.262)	(0.865, 1.345)
Homemaker/student	0.617***	0.521***	0.662***	0.630***	0.957	0.855
	(0.550, 0.693)	(0.430, 0.632)	(0.571, 0.768)	(0.556, 0.714)	(0.665, 1.378)	(0.460, 1.587)
Other	1.160***	1.113	1.206**	1.085	1.420***	1.431*
	(1.063, 1.266)	(0.977, 1.268)	(1.075, 1.354)	(0.974, 1.209)	(1.211, 1.664)	(1.027, 1.994)
Constant	0.540***	0.840	0.287***	0.401***	0.496	5.889**
	(0.424, 0.687)	(0.608, 1.160)	(0.202, 0.408)	(0.296, 0.544)	(0.210, 1.172)	(1.603, 21.633)
Year Fixed Effect	Y	Y	Y	Y	Y	Y
Month Fixed Effect	Y	Y	Y	Y	Y	Y
State Fixed Effect	Y	Y	Y	Y	Y	Y

Observations	535,643	259,190	276,453	176,055	157,014	202,574

Note: Models were estimated using the complex survey weights entailing the sampling frame of the BRFSS, adjusted for the pooled structure of data. 95% confidence intervals are in parenthesis. *** p < 0.001, ** p < 0.01, * p < 0.05. WDS refers to widowed/divorced/separated.

Table A.9. Adjusted odds ratios of heavy drinking

	All	Male	Female	Age 18-49	Age 50-64	Age 65+
**CKMS stages**						
Stage 0	Ref.	Ref.	Ref.	Ref.	Ref.	Ref.
Stage 1	0.916*	1.054	0.890*	0.934	0.763***	0.884
	(0.843, 0.996)	(0.923, 1.205)	(0.799, 0.992)	(0.839, 1.039)	(0.660, 0.881)	(0.700, 1.116)
Stage 2/3	1.105*	1.410***	0.961	1.144*	0.946	0.934
	(1.016, 1.202)	(1.231, 1.614)	(0.862, 1.071)	(1.015, 1.290)	(0.832, 1.076)	(0.778, 1.123)
Stage 4	0.812**	1.101	0.606***	1.042	0.674***	0.723**
	(0.714, 0.924)	(0.915, 1.325)	(0.501, 0.734)	(0.800, 1.358)	(0.554, 0.820)	(0.575, 0.909)
**Demography**						
Age	0.986***	0.985***	0.987***	1.001	0.979***	0.947***
	(0.983, 0.989)	(0.981, 0.990)	(0.984, 0.991)	(0.995, 1.007)	(0.968, 0.991)	(0.933, 0.961)
Female	1.103***			1.118**	1.082	1.117
	(1.045, 1.165)			(1.031, 1.212)	(0.985, 1.188)	(0.993, 1.258)
**Race & Ethnicity**						
White	Ref.	Ref.	Ref.	Ref.	Ref.	Ref.
Black	0.635***	0.631***	0.632***	0.745***	0.479***	0.457***
	(0.572, 0.705)	(0.543, 0.733)	(0.545, 0.734)	(0.651, 0.854)	(0.383, 0.600)	(0.362, 0.578)
Hispanic	0.600***	0.638***	0.550***	0.657***	0.463***	0.547*
	(0.531, 0.678)	(0.547, 0.744)	(0.452, 0.670)	(0.566, 0.763)	(0.370, 0.580)	(0.330, 0.907)
Asian	0.336***	0.393***	0.288***	0.349***	0.277***	0.428*
	(0.278, 0.405)	(0.301, 0.513)	(0.221, 0.376)	(0.281, 0.434)	(0.194, 0.396)	(0.215, 0.854)
Other	0.815***	0.857	0.761**	0.822**	0.802	0.800
	(0.726, 0.914)	(0.735, 1.000)	(0.639, 0.905)	(0.711, 0.950)	(0.641, 1.003)	(0.576, 1.112)
**Marital status**						
Married	Ref.	Ref.	Ref.	Ref.	Ref.	Ref.
WDS	1.164***	1.318***	1.081	1.360***	1.160**	1.090
	(1.084, 1.250)	(1.191, 1.460)	(0.979, 1.193)	(1.201, 1.540)	(1.037, 1.297)	(0.953, 1.247)
Never married	1.060	1.004	1.165**	1.253***	0.926	1.041
	(0.976, 1.151)	(0.893, 1.130)	(1.039, 1.307)	(1.122, 1.398)	(0.797, 1.075)	(0.805, 1.345)
Other	1.254***	1.252**	1.280**	1.295***	1.605***	1.747***
	(1.116, 1.409)	(1.055, 1.486)	(1.095, 1.496)	(1.118, 1.500)	(1.285, 2.004)	(1.262, 2.419)
**Education**						
College degree	Ref.	Ref.	Ref.	Ref.	Ref.	Ref.
≤ High school	1.248***	1.662***	0.913	1.332***	1.379***	0.963
	(1.164, 1.337)	(1.514, 1.825)	(0.820, 1.016)	(1.201, 1.478)	(1.226, 1.551)	(0.841, 1.102)
Some college	1.185***	1.332***	1.106*	1.268***	1.159**	1.016
	(1.113, 1.261)	(1.220, 1.453)	(1.011, 1.210)	(1.155, 1.392)	(1.044, 1.286)	(0.899, 1.148)
**Income**						
High income	Ref.	Ref.	Ref.	Ref.	Ref.	Ref.
Low income	0.609***	0.712***	0.545***	0.611***	0.639***	0.618***
	(0.558, 0.664)	(0.629, 0.806)	(0.483, 0.614)	(0.535, 0.696)	(0.554, 0.737)	(0.510, 0.749)
Middle income	0.794***	0.866**	0.728***	0.840**	0.781***	0.751***
	(0.746, 0.846)	(0.791, 0.948)	(0.666, 0.795)	(0.757, 0.932)	(0.704, 0.867)	(0.665, 0.849)
**Employment**						
Employed	Ref.	Ref.	Ref.	Ref.	Ref.	Ref.
Retired	0.886*	0.839**	0.957	0.941	1.312***	0.953
	(0.807, 0.973)	(0.735, 0.958)	(0.838, 1.093)	(0.532, 1.666)	(1.129, 1.524)	(0.788, 1.152)
Homemaker/student	0.568***	0.424***	0.651***	0.589***	0.641***	0.717
	(0.501, 0.644)	(0.329, 0.547)	(0.563, 0.754)	(0.504, 0.689)	(0.499, 0.823)	(0.454, 1.133)
Other	0.869*	0.946	0.809**	0.805**	0.859	1.086
	(0.779, 0.969)	(0.808, 1.106)	(0.695, 0.942)	(0.694, 0.935)	(0.731, 1.010)	(0.690, 1.712)
Constant	0.141***	0.110***	0.158***	0.065***	0.250***	3.312*
	(0.111, 0.178)	(0.078, 0.153)	(0.115, 0.218)	(0.045, 0.092)	(0.118, 0.528)	(1.070, 10.252)
Year Fixed Effect	Y	Y	Y	Y	Y	Y
Month Fixed Effect	Y	Y	Y	Y	Y	Y
State Fixed Effect	Y	Y	Y	Y	Y	Y

Observations	526,760	254,371	272,389	172,685	154,381	199,694

Note: Models were estimated using the complex survey weights entailing the sampling frame of the BRFSS, adjusted for the pooled structure of data. 95% confidence intervals are in parenthesis. *** $p < 0.001$, ** $p < 0.01$, * $p < 0.05$. WDS refers to widowed/divorced/separated.

Table A.10. Female-male differences in predictive margins: average marginal effects

	All	Age 18-49	Age 50-64	Age 65+
**A. Current smoking**				
Stage 0	-0.040***	-0.042***	-0.072***	-0.082***
	(-0.051, -0.029)	(-0.056, -0.028)	(-0.096, -0.047)	(-0.113, -0.051)
Stage 1	-0.024***	-0.024***	-0.024*	-0.031**
	(-0.031, -0.017)	(-0.033, -0.015)	(-0.042, -0.006)	(-0.051, -0.011)
Stage 2/3	-0.012***	-0.021***	0.003	-0.007
	(-0.018, -0.006)	(-0.031, -0.010)	(-0.006, 0.013)	(-0.016, 0.001)
Stage 4	-0.009	0.004	0.006	-0.017*
	(-0.021, 0.003)	(-0.034, 0.041)	(-0.016, 0.027)	(-0.031, -0.003)
**B. Current vaping**				
Stage 0	-0.014***	-0.025***	-0.008	-0.008
	(-0.021, -0.007)	(-0.037, -0.013)	(-0.018, 0.003)	(-0.018, 0.003)
Stage 1	-0.009***	-0.015***	-0.002	-0.003
	(-0.013, -0.004)	(-0.023, -0.007)	(-0.009, 0.006)	(-0.008, 0.002)
Stage 2/3	-0.003	-0.012	0.004	0.000
	(-0.009, 0.002)	(-0.025, 0.000)	(-0.001, 0.009)	(-0.002, 0.003)
Stage 4	0.004	-0.012	0.008	0.002
	(-0.013, 0.020)	(-0.061, 0.038)	(-0.008, 0.024)	(-0.004, 0.007)
**C. Heavy drinking**				
Stage 0	0.024***	0.028***	0.012	0.026**
	(0.016, 0.031)	(0.017, 0.039)	(-0.004, 0.028)	(0.010, 0.041)
Stage 1	0.010***	0.010**	0.014**	0.007
	(0.005, 0.015)	(0.003, 0.017)	(0.005, 0.023)	(-0.007, 0.020)
Stage 2/3	-0.001	-0.007	0.003	0.004
	(-0.006, 0.004)	(-0.017, 0.003)	(-0.005, 0.011)	(-0.003, 0.011)
Stage 4	-0.015**	-0.042**	-0.016*	-0.003
	(-0.024, -0.006)	(-0.070, -0.013)	(-0.029, -0.003)	(-0.014, 0.008)

Note: Standard errors are estimated using Delta method. 95% confidence intervals are in parenthesis. *** $p < 0.001$, ** $p < 0.01$, * $p < 0.05$.

Figure A.1. Crude and adjusted predictive margins of current smoking by CKMS stages.

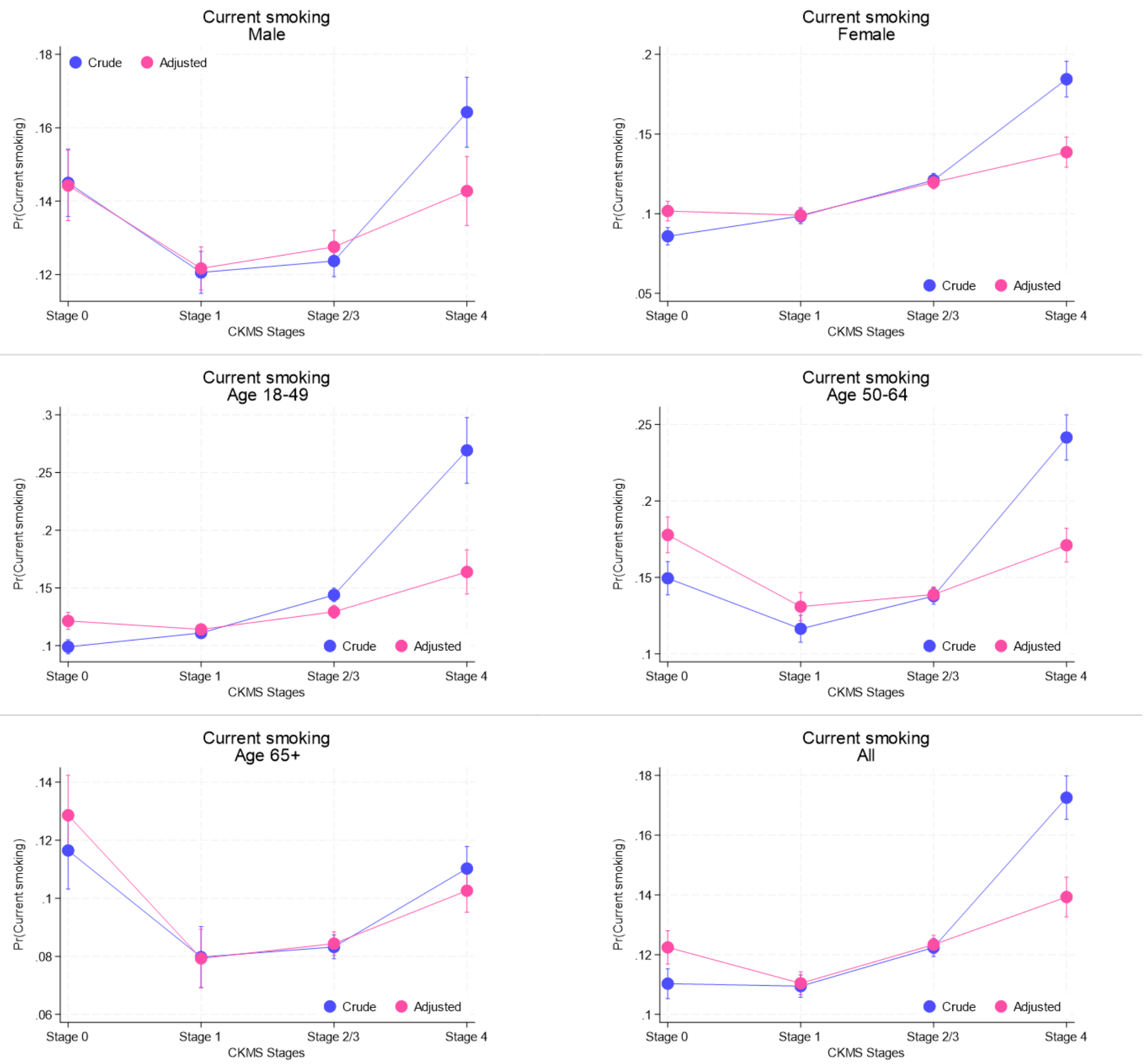

Note: Models were estimated using the complex survey weights entailing the sampling frame of the BRFSS, adjusted for the pooled structure of data. Adjustments were made for age, sex, race and ethnicity, marital status, educational attainment, income, employment status, survey year fixed effects, interview month fixed effects, and state of residence fixed effects.

Figure A.2. Crude and adjusted predictive margins of current vaping by CKMS stages.

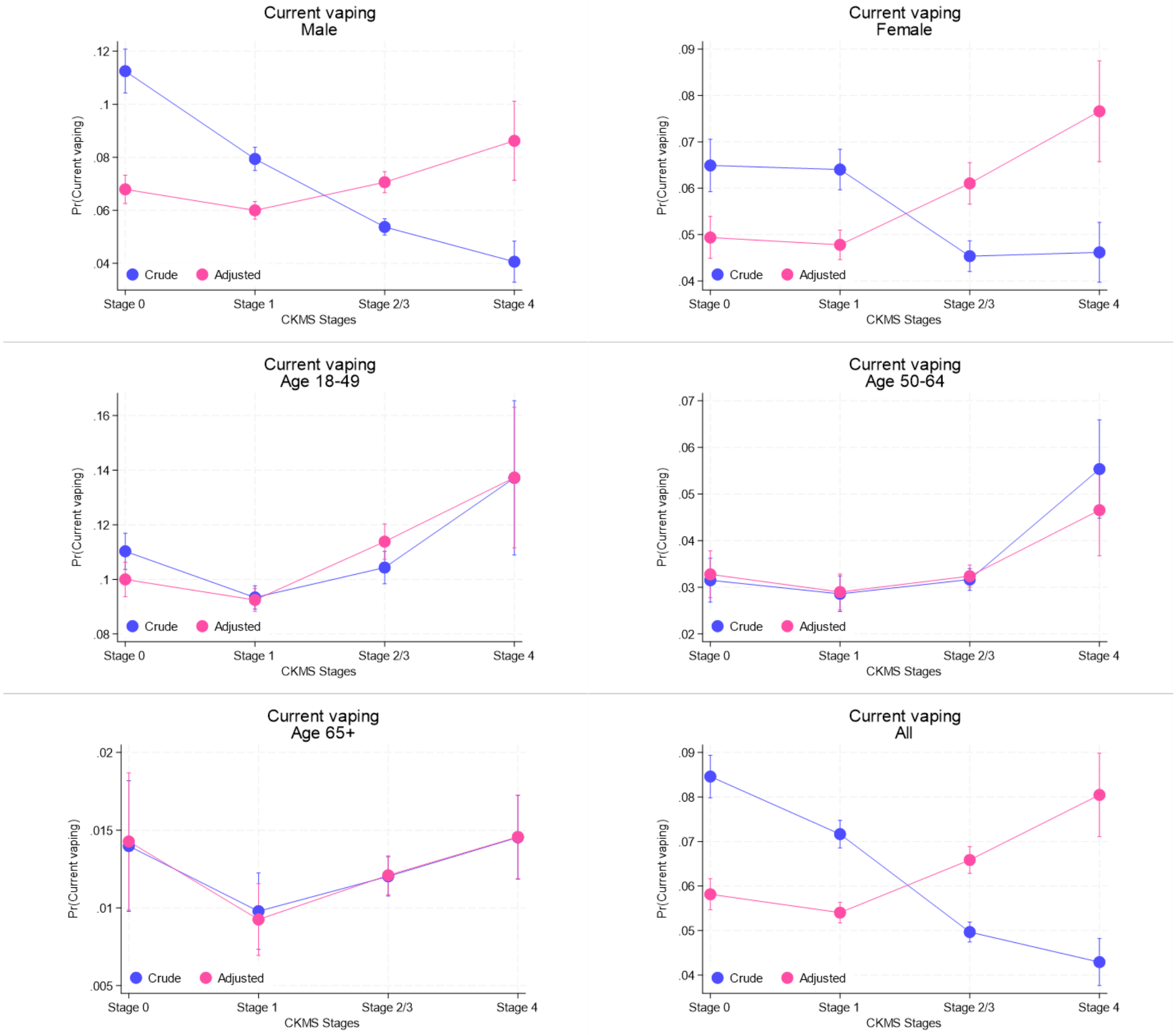

Note: Models were estimated using the complex survey weights entailing the sampling frame of the BRFSS, adjusted for the pooled structure of data. Adjustments were made for age, sex, race and ethnicity, marital status, educational attainment, income, employment status, survey year fixed effects, interview month fixed effects, and state of residence fixed effects.

Figure A.3. Crude and adjusted predictive margins of heavy drinking by CKMS stages.

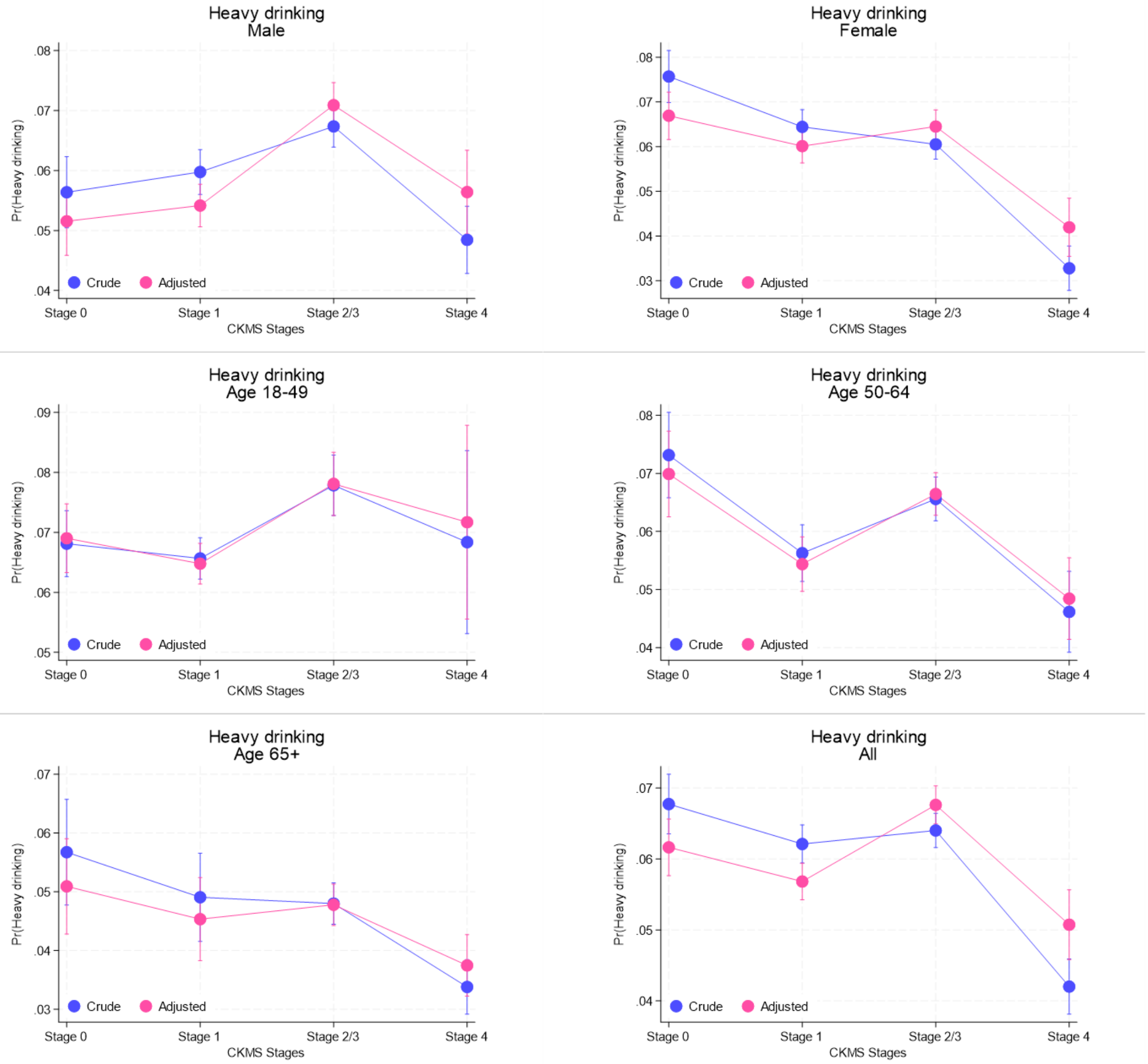

Note: Models were estimated using the complex survey weights entailing the sampling frame of the BRFSS, adjusted for the pooled structure of data. Adjustments were made for age, sex, race and ethnicity, marital status, educational attainment, income, employment status, survey year fixed effects, interview month fixed effects, and state of residence fixed effects.
